# Growth of *Yersinia pseudotuberculosis *in human plasma: impacts on virulence and metabolic gene expression

**DOI:** 10.1186/1471-2180-8-211

**Published:** 2008-12-03

**Authors:** Marie-Laure Rosso, Sylvie Chauvaux, Rodrigue Dessein, Caroline Laurans, Lionel Frangeul, Céline Lacroix, Angèle Schiavo, Marie-Agnès Dillies, Jeannine Foulon, Jean-Yves Coppée, Claudine Médigue, Elisabeth Carniel, Michel Simonet, Michaël Marceau

**Affiliations:** 1 Inserm U801, Lille, F-59019, France; Université Lille II (Faculté de Médecine Henri Warembourg), F-59045 Lille, France; Institut Pasteur de Lille, F-59019 Lille, France; 2 *Yersinia *research Unit, Institut Pasteur, 28 rue du Dr. Roux, F-75724 Paris cedex 15, France; 3 Plate-Forme 4, Institut Pasteur, 28 rue du Dr. Roux, F-75724 Paris cedex 15, France; 4 Plate-Forme 2, Institut Pasteur, 28 rue du Dr. Roux, F-75724 Paris cedex 15, France; 5 Génoscope, 2 rue Gaston Crémieux, CP5706, F-91057 Evry, France; 6 Unité de Prévention et Thérapies Moléculaires des Maladies Humaines, Centre National de Référence de la coqueluche et autres bordetelloses, 28 rue du Dr. Roux, F-75724 Paris cedex 15, France; 7 Fédération de Biologie, Centre Hospitalier de Roubaix, 11-17 Bd Lacordaire, F-59056 Roubaix, France

## Abstract

**Background:**

In man, infection by the Gram-negative enteropathogen *Yersinia pseudotuberculosis *is usually limited to the terminal ileum. However, in immunocompromised patients, the microorganism may disseminate from the digestive tract and thus cause a systemic infection with septicemia.

**Results:**

To gain insight into the metabolic pathways and virulence factors expressed by the bacterium at the blood stage of pseudotuberculosis, we compared the overall gene transcription patterns (the transcriptome) of bacterial cells cultured in either human plasma or Luria-Bertani medium. The most marked plasma-triggered metabolic consequence in *Y. pseudotuberculosis *was the switch to high glucose consumption, which is reminiscent of the acetogenic pathway (known as "glucose overflow") in *Escherichia coli*. However, upregulation of the glyoxylate shunt enzymes suggests that (in contrast to *E. coli*) acetate may be further metabolized in *Y. pseudotuberculosis*. Our data also indicate that the bloodstream environment can regulate major virulence genes (positively or negatively); the *yadA *adhesin gene and most of the transcriptional units of the pYV-encoded type III secretion apparatus were found to be upregulated, whereas transcription of the pH6 antigen locus was strongly repressed.

**Conclusion:**

Our results suggest that plasma growth of *Y. pseudotuberculosis *is responsible for major transcriptional regulatory events and prompts key metabolic reorientations within the bacterium, which may in turn have an impact on virulence.

## Background

The Gram-negative bacterium *Y. pseudotuberculosis *is a human enteropathogen which is able to cross the intestinal mucosa through the M cells in Peyer's patches and thus infect the underlying tissues (causing ileitis and mesenteric lymphadenitis). However, in elderly or debilitated individuals (those suffering from malignancies, immunodeficiencies, chronic liver diseases or diabetes mellitus, for example), the organism frequently gains access to the bloodstream and can cause an often fatal septicemia [1,2]. Known *Y. pseudotuberculosis *virulence genes are transcriptionally regulated by temperature – most probably in order to adapt to the bacterium's life cycle outside and inside the host. Regulation by the omnipresent thermal stimulus can be modulated (via a wide range of mechanisms) by signals such as pH, other ion concentrations and nutrient availability (reviewed in [3]). This allows bacterial pathogens to (i) adapt their gene transcription profiles in response to environmental cues sensed during the course of infection and (ii) express the most appropriate virulence factors at the expense of useless (or even detrimental) ones.

To date, the transcriptional gene regulation occurring when *Y. pseudotuberculosis *enters the human bloodstream has only been inferred indirectly from *in vivo *results in rodent models of infection [4,5] and *in vitro *gene transcription studies. The *in vitro *regulation of certain *Yersinia *virulence loci has mainly been analyzed with respect to single growth parameter changes mimicking the environmental signals known (or assumed) to be detected by bacteria in blood, such as iron scarcity, oxygen tension and pH [6,7]. In the present work, we have adopted an intermediate approach by comparing the overall gene expression profiles of *Y. pseudotuberculosis *grown in human plasma and in Luria-Bertani broth. We then compared the observed variations with those recently published for *Y. pestis *[8], an almost genetically identical pathogen which, however, causes plague – one of the most severe systemic infections in humans and other mammals.

## Results and discussion

The genome of *Y. pseudotuberculosis *strain IP32953 has been recently deciphered: it contains 3,951 coding sequences (CDSs), of which 99 are borne by the virulence-associated plasmid pYV and 43 are carried by a 27-kb cryptic plasmid. Only around 49% of CDSs encode a product with a putative or proven function [9]. To gain insight into the transcriptional regulation of virulence and metabolism genes that takes place when *Y. pseudotuberculosis *enters and multiplies in the bloodstream, we compared the transcriptome of IP32953 grown in human plasma to the one of the same strain grown in Luria-Bertani (LB). To this end, we prepared macroarrays composed of 3,674 PCR fragments of ≈ 400-base pairs (bp), covering 96% of IP32953's CDSs and used them as described elsewhere [8] and in the Methods section. Briefly, in three independent cultures, total RNA was extracted from IP32953 cells grown in LB broth or human plasma, in the exponential or stationary phase and at 28°C or 37°C. Macroarray probing was performed three times with independently retrotranscribed and ^33^P-radiolabeled RNA samples from each of the eight growth combinations. After macroarray imaging, hybridization intensity data were log-transformed and normalized using a simple median normalization method. Relative data have been deposited in the Genoscript database  in accordance with standards of the Microarray Gene Expression Data Society (MGED). An analysis of variance (ANOVA) was carried out independently for each gene, with the three biological factors of variation (medium, temperature and growth phase) as fixed effects. This statistical approach allowed us to evaluate the transcriptional variations induced by each factor for the dataset as a whole. Thus, three ratios (corresponding to each parameter) and associated *p*-values were calculated for each gene. Inter-condition transcriptional differences were considered to be statistically significant if the *p*-value was below 0.05. Representative macroarray hybridization results were confirmed by qRT-PCR on stored RNA samples, using the constitutively expressed *YPTB0775* gene (spot ID YPO3356 and coding for the outer membrane lipoprotein NplD) as a reference (Additional file 1). Since the physiological status of the bacterium during host infection is unknown, we focused our analysis on genes regulated by the temperature and/or the medium in both the exponential and stationary phases. All *Y. pseudotuberculosis *transcriptional variations discussed herein were compared with those of their respective *Y. pestis *orthologs and are summarized in Table 1. *Y. pseudotuberculosis *IP32953 genes regulated at the transcriptional level by growth temperature and/or medium are listed in Tables 2 and 3.

**Table 1:** *Y. pseudotuberculosis *transcriptional variations discussed in this article compared with those recently published for *Y. pestis *[8]

**Locus tags**				**gene transcription fold ratio human plasma/Luria Bertani broth **(*p*-value)			
			
***Y. pseudotuberculosis***	***Y. pestis***	**Gene Name**	**Putative product/function**	***Y. pseudotuberculosis***		***Y. pestis***	
**Iron uptake and storage**							
YPTB1659	YPO1783	*ftnA*	ferritin	**0.180**	(< 0.001)	**0.341**	(0.001)
YPTB0336	YPO0279	*hmuV*	ABC hemin transporter, ATP-binding subunit HmuV	**1.665**	(0.003)	**1.499**	(0.021)
YPTB0338	YPO0281	*hmuT*	ABC transporter, periplasmic hemin-binding protein HmuT	**1.684**	(0.016)	**2.149**	(0.001)
YPTB0339	YPO0282	*hmuS*	possible hemin degradation/transport protein HmuS	**1.577**	(0.022)	**1.984**	(< 0.001)
YPTB0340	YPO0283	*hmuR*	TonB-dependent outer membrane hemin receptor, HmuR	**7.426**	(< 0.001)	**2.028**	(< 0.001)
YPTB0739	YPO3392	*fhuC*	putative ABC type hydroxymate-dependent iron transport ATP binding protein	**1.932**	(0.047)	**1.500**	(0.258)
YPTB0740	YPO3391	*fhuD*	putative ABC type hydroxamate-dependent iron uptake ATP binding protein	**1.836**	(0.021)	**1.174**	(0.486)
YPTB1341	YPO1310	*yiuA*	putative ABC type periplasmic iron siderophore/cobalamin binding protein	**3.085**	(< 0.001)	**1.737**	(0.027)
YPTB1343	YPO1312	*yiuC*	putative siderophore/cobalamin ABC transporter, ATP-binding subunit	**2.468**	(< 0.001)	**1.991**	(0.012)
YPTB1512	YPO1496		putative heme-binding protein	**2.456**	(< 0.001)	**0.921**	(0.639)
YPTB1513	YPO1497		putative ABC transporter ATP-binding protein	**1.877**	(0.009)	**1.408**	(0.113)
YPTB1515	YPO1499		putative membrane protein	**1.521**	(0.045)	**1.018**	(0.921)
YPTB1540	YPO1528	*ysuF*	putative ferric iron reductase	**4.019**	(< 0.001)	**3.137**	(< 0.001)
YPTB1541	YPO1529	*ysuJ*	putative decarboxylase	**2.092**	(< 0.001)	**1.246**	(0.236)
YPTB1543	YPO1531	*ysuH*	putative siderophore biosynthetic enzyme	**1.896**	(0.021)	**1.213**	(0.387)
YPTB1544	YPO1532	*ysuG*	putative siderophore biosynthetic enzyme	**2.403**	(0.005)	**1.830**	(0.006)
YPTB1549	YPO1537	*ysuR*	putative OMR family iron-siderophore receptor	**2.610**	(< 0.001)	**3.273**	(0.001)
YPTB2117	YPO2193	*tonB*	TonB protein	**5.464**	(< 0.001)	**2.563**	(0.001)
YPTB2347	YPO2439	*yfeA*	ABC transporter, periplasmic iron siderophore-binding protein YfeA	**11.88**	(< 0.001)	**10.22**	(< 0.001)
YPTB2348	YPO2440	*yfeB*	ABC chelated iron transporter, ATP-binding subunit YfeB	**3.141**	(< 0.001)	**2.772**	(0.001)
YPTB2349	YPO2441	*yfeC*	ABC chelated iron transporter, permease subunit YfeC	**4.375**	(< 0.001)	**1.817**	(0.017)
YPTB2350	YPO2442	*yfeD*	ABC chelated iron transporter, permease subunit YfeD	**2.103**	(< 0.001)	**1.369**	(0.195)
YPTB2353	YPO2445	*yfeE*	putative yfeABCD locus regulator	**1.527**	(0.008)	**2.085**	(0.001)
YPTB3263	YPO0989	*iucA*	possible siderophore biosynthesis protein, IucA familly	**3.789**	(< 0.001)	**2.059**	(0.033)
YPTB3265	YPO0992	*iucC*	putative siderophore biosynthesis protein IucC	**2.600**	(< 0.001)	**0.920**	(0.711)
YPTB3266	YPO0993	*iucD*	putative siderophore biosynthesis protein IucD	**2.151**	(0.002)	**0.631**	(0.036)
YPTB3298	YPO1011		putative TonB-dependent O-M, iron siderophore receptor/tranporter	**2.150**	(0.005)	**1.845**	(0.018)
YPTB3383	YPO0682	*exbB*	possible MotA/TolQ/ExbB proton channel family protein	**4.165**	(< 0.001)	**3.021**	(< 0.001)
YPTB3382	YPO0683	*exbD*	pExbD/TolR-family transport protein	**9.811**	(< 0.001)	**3.576**	(< 0.001)
YPTB3701	YPO0205	*bfd*	putative bacterioferritin-associated ferredoxin	**1.857**	(0.002)	**3.195**	(< 0.001)
YPTB3700	YPO0206	*bfr*	bacterioferritin	**1.153**	(0.255)	**4.483**	(< 0.001)
YPTB3767	YPO0133	*feoA*	conserved hypothetical protein	**1.510**	(0.046)	**1.081**	(0.648)
YPTB3769	YPO0131	*feoC*	conserved hypothetical protein	**1.900**	(0.001)	**1.964**	(0.002)
YPTB3857	YPO4022		putative ABC transporter, periplasmic iron siderophore ferrichrome binding protein	**3.127**	(< 0.001)	**2.157**	(< 0.001)
YPTB3858	YPO4023		putative ABC iron siderophore transporter, permease subunit	**2.236**	(0.001)	**1.214**	(0.529)
YPTB3860	YPO4025		putative ABC iron siderophore transporter, ATP-binding subunit	**2.216**	(0.001)	**1.417**	(0.041)
YPTB1246	YPO1207	*katA*	putative catalase	**0.324**	(< 0.001)	**0.556**	(0.002)
YPTB0811	YPO3319	*katY*	putative catalase-hydroperoxidase HPI I	**0.603**	(0.009)	**0.717**	(0.342)
**Biotin operon**							
YPTB1181	YPO1150	*bioA*	putative adenosylmethionine-8-amino-7-oxononanoate aminotransferase	**3.377**	(< 0.001)	**2.512**	(0.002)
YPTB1183	YPO1152	*bioF*	putative 8-amino-7-oxononanoate synthase	**3.800**	(< 0.001)	**2.595**	(< 0.001)
YPTB1184	YPO1153	*bioC*	putative biotin synthesis protein BioC	**1.784**	(0.004)	**1.351**	(0.374)
YPTB1185	YPO1154	*bioD*	putative dethiobiotin synthetase	**2.499**	(0.002)	**1.780**	(0.026)
**Superoxyde dismutases**							
YPTB3925	YPO4061	*sodA*	putative superoxide dismutase [Mn]	**3.101**	(< 0.001)	**1.796**	(0.049)
YPTB2299	YPO2386	*sodB*	superoxide dismutase [Fe]	**0.090**	(< 0.001)	**0.326**	(< 0.001)
**Ribonucleotides reductases (RNR)**							
YPTB2956	YPO2650	*nrdI*	probable NrdI protein homologue	**2.842**	(0.001)	**8.784**	(< 0.001)
YPTB2957	YPO2649	*nrdE*	putative ribonucleoside-diphosphate reductase 2 alpha chain	**4.745**	(< 0.001)	**9.007**	(< 0.001)
YPTB2958	YPO2648	*nrdF*	putative ribonucleoside-diphosphate reductase 2 beta chain	**2.672**	(0.002)	**2.142**	(0.011)
YPTB2955	YPO2651	*nrdH*	putative glutaredoxin	**1.407**	(0.152)	**3.439**	(< 0.001)
YPTB1254	YPO1214	*nrdA*	putative ribonucleoside-diphosphate reductase 1 alpha chain	**0.329**	(< 0.001)	**0.668**	(0.085)
YPTB1253	YPO1213	*nrdB*	putative ribonucleoside-diphosphate reductase 1 beta chain	**0.815**	(0.351)	**0.579**	(0.020)
YPTB0519	YPO3454	*nrdD*	putative anaerobic ribonucleoside-triphosphate reductase	**0.614**	(0.025)	**0.373**	(< 0.001)
YPTB0518	YPO3455	*nrdG*	putative anaerobic ribonucleotide reductase activating protein	**0.440**	(0.001)	**0.508**	(0.019)
**Mannose and glucose uptake**							
YPTB1634	YPO1758	*manX*	probable PTS system, mannose-specific IIAB component	**2.673**	(< 0.001)	**3.234**	(< 0.001)
YPTB1633	YPO1757	*manY*	probable PTS system, mannose-specific IIC component	**1.991**	(0.006)	**2.201**	(< 0.001)
YPTB1632	YPO1756	*manZ*	probable PTS system, mannose-specific IID component	**3.084**	(< 0.001)	**3.676**	(< 0.001)
YPTB2463	YPO1608	*ptsG*,	putative PTS system, glucose-specific IIBC component	**4.033**	(< 0.001)	**1.708**	(0.027)
YPTB2715	YPO2993	*ptsH*	probable PTS system, phosphocarrier protein	**1.697**	(0.002)	**1.422**	(0.109)
YPTB2716	YPO2994	*ptsI*	putative PTS sytem, enzyme I component	**1.946**	(< 0.001)	**1.351**	(0.038)
YPTB2717	YPO2995	*crr*	putative PTS system, glucose-specific IIA component, permease	**1.611**	(0.002)	**1.440**	(0.117)
**Sugar metabolism**							
YPTB0074	YPO0078	*pfkA*	putative 6-phosphofructokinase	**1.594**	(0.017)	**1.430**	(0.073)
YPTB3195	YPO0920	*fbaA, fba, fda*	possible fructose-bisphosphate aldolase class II	**1.325**	(0.049)	**1.504**	(0.104)
YPTB3196	YPO0921	*pgk*	putative phosphoglycerate kinase	**1.365**	(0.024)	**1.365**	(0.099)
YPTB1166	YPO1133	*gpmA, gpm*	putative phosphoglycerate mutase 1	**3.179**	(< 0.001)	**3.650**	(< 0.001)
YPTB2047	YPO2064	*pykA*	putative pyruvate kinase II	**0.486**	(0.001)	**0.604**	(0.013)
YPTB2306	YPO2393	*pykF*	probable pyruvate kinase I	**2.282**	(< 0.001)	**1.322**	(0.185)
YPTB3762	YPO0138	*pck*	putative phosphoenolpyruvate carboxykinase [ATP]	**0.395**	(0.001)	**1.213**	(0.460)
YPTB2103	YPO2180	*adhE, ana*	putative aldehyde-alcohol dehydrogenase	**2.116**	(0.001)	**3.383**	(< 0.001)
YPTB0460	YPO3516	*mdh*	putative malate dehydrogenase	**0.665**	(0.010)	**0.394**	(< 0.001)
YPTB0796	YPO3335	*fumA, fumB*	putative fumarase A fumarate hydratase class I, aerobic isozyme	**0.351**	(< 0.001)	**0.501**	(0.020)
YPTB0413	YPO0360	*frdA*	putative fumarate reductase flavoprotein subunit	**0.156**	(< 0.001)	**0.299**	(< 0.001)
YPTB0412	YPO0359	*frdB*	putative fumarate reductase iron-sulfur protein	**0.127**	(< 0.001)	**0.217**	(< 0.001)
YPTB0411	YPO0358	*frdC*	putative fumarate reductase hydrophobic protei	**0.248**	(< 0.001)	**0.403**	(< 0.001)
YPTB0410	YPO0357	*frdD*	putative fumarate reductase hydrophobic protein	**0.393**	(0.001)	**0.372**	(< 0.001)
YPTB1145	YPO1111	*sdhA*	putative succinate dehydrogenase flavoprotein subunit	**0.497**	(< 0.001)	**0.124**	(< 0.001)
YPTB1144	YPO1110	*sdhD*	putative succinate dehydrogenase hydrophobic membrane anchor protein	**0.501**	(< 0.001)	**0.165**	(< 0.001)
YPTB1143	YPO1109	*sdhC*	putative succinate dehydrogenase cytochrome b-556 subunit	**0.553**	(0.016)	**0.241**	(0.001)
YPTB1146	YPO1112	*sdhB*	putative succinate dehydrogenase iron-sulfur protein	**0.592**	(0.004)	**0.196**	(< 0.001)
YPTB1149	YPO1115	*sucC*	putative succinyl-CoA synthetase beta chain	**0.419**	(< 0.001)	**0.205**	(< 0.001)
YPTB1150	YPO1116	*sucD*	putative succinyl-CoA synthetase alpha chain	**0.525**	(< 0.001)	**0.236**	(0.001)
YPTB1148	YPO1114	*sucB*	putative dihydrolipoamide succinyltransferase component	**0.610**	(0.014)	**0.267**	(0.003)
YPTB1147	YPO1113	*sucA*	putative 2-oxoglutarate dehydrogenase E1 component	**0.516**	(0.007)	**0.193**	(< 0.001)
YPTB0716	YPO3415	*acnB*	putative aconitate hydratase 2	**0.510**	(< 0.001)	**0.319**	(< 0.001)
YPTB3656	YPO3725	*aceA, icl*	isocitrate lyase	**2.068**	(0.003)	**1.298**	(0.319)
YPTB3657	YPO3726	*aceB, mas*	malate synthase A	**1.875**	(0.053)	**1.089**	(0.710)
YPTB2222	YPO2300	*fnr, nirR*	putative fumarate and nitrate reduction regulatory protein	**0.699**	(0.002)	**0.877**	(0.621)
YPTB0601	YPO0458	*arcA*	probable response regulator (OmpR family)	**0.464**	(0.001)	**0.705**	(0.036)
**Porins**							
YPTB1964	YPO1411	*ompC2*	putative outer membrane protein C2, porin	**0.835**	(0.447)	**1.827**	(0.001)
YPTB1261	YPO1222	*ompC*	putative outer membrane protein C, porin	**0.285**	(< 0.001)	**0.659**	(0.013)
YPTB1453	YPO1435	*ompA*	putative outer membrane porin A protein	**1.085**	(0.372)	**0.587**	(0.070)
**Chromosomal virulence factors**							
YPTB1668	YPO3944	*inv*	putative invasin	**0.501**	(< 0.001)	**0.993**	(0.976)
YPTB1334	YPO1303	*psaA*	pH 6 antigen precursor		(< 0.001)	**0.367**	(0.154)
YPTB1335	YPO1304	*psaB*	chaperone protein PsaB precursor	**0.216**	(< 0.001)	**0.700**	(0.214)
YPTB1332	YPO1301	*psaE*	putative regulatory protein	**1.987**	(0.002)	**2.619**	(< 0.001)
**pYV-encoded virulence factors – Type Three Secretion System**							
pYV0062	YPCD1.36c	*yscX*	YscX, putative type III secretion protein	**1.629**	(0.009)	**0.873**	(0.444)
pYV0014	YPCD1.89		possible transposase remnant (pseudogene)	**1.789**	(0.025)	**0.786**	(0.899)
pYV0076	YPCD1.49	*lcrF*	LcrF, VirF; putative thermoregulatory protein	**1.441**	(0.042)	**1.311**	(0.216)
pYV0058	YPCD1.32c	*lcrG*	LcrG, putative Yop regulator	**1.713**	(0.014)	**0.941**	(0.853)
pYV0056	YPCD1.30c	*lcrH, sycD*	LcrH, SycD; low calcium response protein H	**3.522**	(< 0.001)	**0.898**	(0.480)
pYV0059	YPCD1.33c	*lcrR*	LcrR, hypothetical protein	**1.469**	(0.001)	**0.700**	(0.129)
pYV0057	YPCD1.31c	*lcrV*	LcrV, putative V antigen, antihost protein/regulator	**1.904**	(< 0.001)	**1.302**	(0.051)
pYV0024	YPCD1.05c	*sycE*	SycE, yerA; putative yopE chaperone	**5.781**	(< 0.001)	**1.344**	(0.165)
pYV0020	YPCD1.95c	*sycH*	SycH, putative yopH targeting protein	**5.088**	(< 0.001)	**2.029**	(0.002)
pYV0017	YPCD1.91		putative resolvase	**0.341**	(< 0.001)	**1.152**	(0.705)
pYV0075	YPCD1.48	*virG*	VirG; putative Yop targeting lipoprotein	**1.957**	(0.015)	**1.544**	(0.023)
pYV0055	YPCD1.29c	*yopB*	YopB, putative Yop targeting protein	**2.816**	(< 0.001)	**0.956**	(0.758)
pYV0054	YPCD1.28c	*yopD*	YopD, putative Yop negative regulation/targeting component	**2.933**	(< 0.001)	**0.950**	(0.833)
pYV0047	YPCD1.26c	*yopM*	YopM, putative targeted effector protein	**3.467**	(< 0.001)	**1.044**	(0.845)
pYV0065	YPCD1.39c	*yopN*	YopN, LcrE; putative membrane-bound Yop targeting protein	**2.612**	(< 0.001)	**0.878**	(0.475)
pYV0078	YPCD1.51		hypothetical protein	**3.037**	(0.002)	**1.056**	(0.716)
pYV0079	YPCD1.52	*yscC*	YscC, putative type III secretion protein	**2.383**	(0.001)	**0.715**	(0.221)
pYV0080	YPCD1.53	*yscD*	YscD, putative type III secretion protein	**2.541**	(< 0.001)	**0.715**	(0.095)
pYV0081	YPCD1.54	*yscE*	YscE, putative type III secretion protein	**2.710**	(0.001)	**0.543**	(0.056)
pYV0082	YPCD1.55	*yscF*	YscF, putative type III secretion protein	**2.116**	(0.017)	**0.616**	(0.135)
pYV0083	YPCD1.56	*yscG*	YscG, putative type III secretion protein	**2.462**	(< 0.001)	**0.905**	(0.595)
pYV0085	YPCD1.58	*yscI*	YscI, LcrO; putative type III secretion protein	**1.864**	(0.003)	**1.024**	(0.906)
pYV0089	YPCD1.62	*yscM, lcrQ*	YscM, LcrQ, putative type III secretion regulatory	**0.678**	(0.036)	**0.755**	(0.052)
pYV0067	YPCD1.40		putative Yops secretion ATP synthase	**2.855**	(< 0.001)	**0.971**	(0.886)
pYV0068	YPCD1.41	*yscO*	YscO, putative type III secretion protein	**3.087**	(< 0.001)	**0.974**	(0.913)
pYV0070	YPCD1.43	*yscQ*	YscQ, putative type III secretion protein	**1.635**	(0.026)	**1.214**	(0.430)
pYV0071	YPCD1.44	*yscR*	YscR, putative Yop secretion membrane protein	**1.948**	(0.012)	**1.296**	(0.269)
pYV0072	YPCD1.45	*yscS*	YscS, putative type III secretion protein	**2.331**	(< 0.001)	**1.218**	(0.280)
**pYV-encoded virulence factors – Others**							
pYV0013	YPCD1.88c	*yadA*	YadA, *Yersinia *adhesion	**13.52**	(< 0.001)	**1.040**	(0.861)

**Table 2:** *Y. pseudotuberculosis *IP32953 chromosomal genes (sorted by COG class [28]) that are transcriptionally regulated by growth medium and/or temperature.

**COG class**	**Gene designation**	**Genoscript spot ID**	**Gene product/function**	**Fold ratio in gene transcription **(*p*-value)			
				
				**Human plasma/Luria Bertani Broth**		**37°C/28°C**	
**C: energy production and conversion**							
	YPTB0086 *(glpK)*	YPO0090	glycerol kinase	**0.437**	(0.002)		
	YPTB0108 *(ppc)*	YPO3929	phosphoenolpyruvate carboxylase			**0.678**	(0.045)
	YPTB0118	YPO3917	putative pyridine nucleotide-disulphide oxidoreductase			**1.559**	(0.016)
	YPTB0211 *(glpC)*	YPO3824	anaerobic glycerol-3-phosphate dehydrogenase subunit C	**0.49**	(0.003)		
	YPTB0374 *(qor)*	YPO0319	quinone oxidoreductase	**1.393**	(0.04)		
	YPTB0410 *(frdD)*	YPO0357	fumarate reductase hydrophobic protein	**0.393**	(0.001)		
	YPTB0411 *(frdC)*	YPO0358	fumarate reductase hydrophobic protein	**0.248**	(< 0.001)		
	YPTB0412 *(frdB)*	YPO0359	fumarate reductase iron-sulfur protein	**0.128**	(< 0.001)		
	YPTB0413 *(frdA)*	YPO0360	fumarate reductase flavoprotein subunit	**0.157**	(< 0.001)		
	YPTB0460 *(mdh)*	YPO3516	malate dehydrogenase	**0.665**	(0.01)	**0.543**	(< 0.001)
	YPTB0714 *(aceF)*	YPO3418	pyruvate dehydrogenase. dihydrolipoyltransacetylase component			**0.676**	(0.03)
	YPTB0715 *(lpdA)*	YPO3417	dihydrolipoamide dehydrogenase component of pyruvate dehydrogenase complex			**0.657**	(0.01)
	YPTB0716 *(acnB)*	YPO3415	aconitate hydratase 2	**0.51**	(< 0.001)	**0.492**	(< 0.001)
	YPTB0796 *(fumA)*	YPO3335	fumarate hydratase. class I	**0.351**	(< 0.001)	**1.518**	(0.049)
	YPTB0887 *(nqrA)*	YPO3240	NADH-ubiquinone oxidoreductase subunit A	**0.328**	(< 0.001)		
	YPTB0888 *(nqrB)*	YPO3239	NADH-ubiquinone oxidoreductase subunit B	**0.58**	(0.002)		
	YPTB0889 *(nqrC)*	YPO3238	Na+-translocating NADH-quinone reductase subunit c	**0.551**	(0.021)		
	YPTB0892 *(nqrF)*	YPO3235	NADH-uniquinone oxidoreductase subunit F	**0.661**	(0.044)		
	YPTB0895	YPO3232	putative exported protein			**0.6**	(0.008)
	YPTB0949 *(cyoD)*	YPO3167	cytochrome O ubiquinol oxidase subunit CyoD			**0.534**	(0.001)
	YPTB0952 *(cyoA)*	YPO3164	cytochrome O ubiquinol oxidase subunit II	**0.537**	(< 0.001)	**0.516**	(< 0.001)
	YPTB1125 *(fldA)*	YPO2635	flavodoxin 1			**0.722**	(0.033)
	YPTB1143 *(sdhC)*	YPO1109	succinate dehydrogenase cytochrome b-556 subunit	**0.552**	(0.015)	**0.622**	(0.045)
	YPTB1144 *(sdhD)*	YPO1110	succinate dehydrogenase hydrophobic membrane anchor protein	**0.5**	(< 0.001)	**0.654**	(0.014)
	YPTB1145 *(sdhA)*	YPO1111	succinate dehydrogenase flavoprotein subunit	**0.497**	(< 0.001)		
	YPTB1146 *(sdhB)*	YPO1112	succinate dehydrogenase iron-sulfur protein	**0.592**	(0.003)		
	YPTB1147 *(sucA)*	YPO1113	2-oxoglutarate dehydrogenase E1 component	**0.515**	(0.006)		
	YPTB1148 *(sucB)*	YPO1114	dihydrolipoamide succinyltransferase component of 2-oxoglutarate dehydro	**0.61**	(0.013)	**0.671**	(0.04)
	YPTB1149 *(sucC)*	YPO1115	succinyl-CoA synthetase beta chain	**0.419**	(< 0.001)		
	YPTB1150 *(sucD)*	YPO1116	succinyl-CoA synthetase alpha chain	**0.524**	(< 0.001)		
	YPTB1151 *(cydA)*	YPO1117	cytochrome D ubiquinol oxidase subunit I	**0.442**	(< 0.001)		
	YPTB1152 *(cydB)*	YPO1118	cytochrome D ubiquinol oxidase subunit II	**0.565**	(0.004)		
	YPTB1408 *(pflB)*	YPO1383	formate acetyltransferase 1	**0.462**	(< 0.001)	**1.911**	(< 0.001)
	YPTB1723 *(putA)*	YPO1851	bifunctional PutA protein [includes: proline dehydrogenase and delta-1-p	**0.643**	(< 0.001)	**0.642**	(< 0.001)
	YPTB1945	YPO1947	putative thioredoxin	**1.646**	(0.002)		
	YPTB2012	YPO2028	putative exported protein			**0.6**	(0.008)
	YPTB2017	YPO2035	hypothetical protein	**1.694**	(0.045)		
	YPTB2089	YPO2163	putative nitroreductase	**2.095**	(< 0.001)		
	YPTB2103 *(adhE)*	YPO2180	aldehyde-alcohol dehydrogenase	**2.115**	(< 0.001)		
	YPTB2165	YPO2244	Fe-S binding NADH dehydrog. (pseudogene. F/S)			**2.157**	(< 0.001)
	YPTB2224 *(pntB)*	YPO2302	NAD(P) transhydrogenase subunit beta			**1.502**	(0.037)
	YPTB2248 *(ldhA)*	YPO2329	D-lactate dehydrogenase	**0.615**	(0.006)		
	YPTB2253 *(nifJ)*	YPO2334	putative pyruvate-flavodoxin oxidoreductase			**1.697**	(0.014)
	YPTB2427 *(icdA)*	YPO1641	isocitrate dehydrogenase [NADP]			**0.632**	(0.013)
	YPTB2529	YPO2492	putative dioxygenase beta subunit	**0.535**	(0.02)		
	YPTB2578 *(nuoK)*	or3622	NADH dehydrogenase i chain k	**0.642**	(0.001)		
	YPTB2581 *(nuoH)*	YPO2549	NADH dehydrogenase I chain H	**0.745**	(0.023)		
	YPTB2585 *(nuoD)*	YPO2553	NADH dehydrogenase I chain C/D	**0.573**	(0.003)		
	YPTB2587 *(nuoA)*	YPO2555	NADH dehydrogenase I chain A	**0.62**	(< 0.001)		
	YPTB2597 *(ackA)*	YPO2566	acetate kinase	**0.652**	(0.001)		
	YPTB2598 *(pta)*	YPO2567	phosphate acetyltransferase	**0.667**	(0.017)		
	YPTB2689 *(dmsB)*	YPO2966	putative dimethyl sulfoxide reductase chain B protein	**0.598**	(0.031)		
	YPTB2703	YPO2980	putative ion channel protein	**0.565**	(0.01)		
	YPTB2758 *(napC)*	YPO3036	cytochrome C-type protein NapC			**0.636**	(0.015)
	YPTB3469 *(fadH)*	YPO0589	2.4-dienoyl-CoA reductase	**0.465**	(0.002)	**1.713**	(0.024)
	YPTB3539	YPO3694	putative cytochrome			**0.544**	(< 0.001)
	YPTB3592	YPO3637	putative carbohydrate kinase			**1.585**	(0.031)
	YPTB3656 *(aceA)*	YPO3725	isocitrate lyase	**2.069**	(0.002)	**1.963**	(0.004)
	YPTB3762 *(pckA)*	YPO0138	phosphoenolpyruvate carboxykinase [ATP]	**0.395**	(0.001)		
	YPTB3782 *(glpD)*	YPO3937	aerobic glycerol-3-phosphate dehydrogenase	**0.409**	(0.02)		
	YPTB3927 *(fdoG)*	or2536	formate dehydrogenase-O. major subunit	**0.208**	(< 0.001)	**0.712**	(0.029)
	YPTB3928 *(fdoH)*	YPO4057	formate dehydrogenase-O. iron-sulfur subunit	**0.187**	(< 0.001)	**0.698**	(0.03)
	YPTB3929 *(fdoI)*	YPO4056	formate dehydrogenase. cytochrome b556 protein	**0.372**	(< 0.001)	**0.551**	(< 0.001)
	YPTB3967 *(atpD)*	YPO4121	ATP synthase beta subunit protein			**0.679**	(0.025)
	YPTB3968 *(atpG)*	YPO4122	ATP synthase gamma subunit protein			**0.658**	(0.011)
	YPTB3970 *(atpH)*	or2565	ATP synthase delta subunit protein			**0.7**	(0.005)
	YPTB3971 *(atpF)*	YPO4125	ATP synthase subunit B protein			**0.616**	(0.002)
	YPTB3972 *(atpE)*	or2563	ATP synthase subunit C protein			**0.656**	(0.037)
	YPTB3973 *(atpB)*	YPO4127	ATP synthase subunit B protein	**0.667**	(0.004)	**0.732**	(0.021)
**D: cell division and chromosome partitioning**							
	YPTB0222 *(ftsE)*	YPO3813	cell division ATP-binding protein			**0.595**	(0.019)
	YPTB1430 *(mukB)*	YPO1405	cell division protein			**0.62**	(0.006)
	YPTB2923	YPO2686	putative membrane protein	**0.358**	(< 0.001)		
	YPTB3126	or3195	possible bacteriophage protein	**1.616**	(0.029)		
	YPTB3976 *(gidA)*	YPO4130	glucose inhibited division protein A	**0.607**	(0.007)		(0.007)
**E: amino acid transport and metabolism**							
	YPTB0003 *(asnA)*	YPO0003	aspartate-ammonia ligase			**1.531**	(0.042)
	YPTB0024 *(glnA)*	YPO0024	glutamine synthetase	**0.49**	(< 0.001)	**0.448**	(< 0.001)
	YPTB0057 *(tdh)*	YPO0060	threonine 3-dehydrogenase	**0.663**	(0.041)		
	YPTB0066 *(cysE)*	YPO0070	serine acetyltransferase	**1.669**	(0.016)		
	YPTB0106 *(metL)*	YPO0116	bifunctional aspartokinase/homoserine dehydrogenase II	**1.565**	(0.045)		
	YPTB0111 *(argB)*	YPO3925	acetylglutamate kinase	**1.495**	(0.016)		
	YPTB0112 *(argH)*	YPO3924	putative argininosuccinate lyase	**1.779**	(0.007)		
	YPTB0134 *(ilvG)*	YPO3901	acetolactate synthase isozyme II large subunit			**0.627**	(0.039)
	YPTB0203 *(rhtC)*	YPO3832	threonine efflux protein			**0.597**	(0.024)
	YPTB0210 *(glpB)*	YPO3825	putative anaerobic glycerol-3-phosphate dehydrogenase subunit B	**0.554**	(0.01)		
	YPTB0226 *(livK)*	YPO3808	branched-chain amino acid-binding protein	**1.49**	(0.032)		
	YPTB0245	or0170	conserved hypothetical protein	**0.662**	(0.031)		
	YPTB0248 *(metE)*	YPO3788	5-methyltetrahydropteroyltriglutamate – homocystei ne methyltransferase			**2.813**	(0.001)
	YPTB0345	YPO0287	putative methylenetetrahydrofolate reductase	**2.947**	(< 0.001)		
	YPTB0402 *(aspA)*	YPO0348	aspartate ammonia-lyase	**0.224**	(< 0.001)		
	YPTB0407	YPO0353	conserved hypothetical protein			**0.687**	(0.03)
	YPTB0521	YPO3452	putative ABC transporter transporter. ATP-binding protein	**1.679**	(0.029)		
	YPTB0524	YPO3448	(7G) putative extracellular solute-binding protein (pseudogene. F/S)			**1.553**	(0.046)
	YPTB0557	or0391	possible conserved cysteine desulfurase				(< 0.001)
	PTB0602 *(arcA)*	YPO0459	aerobic respiration control protein	**0.465**	(0.001)		
	YPTB0604 *(thrB)*	YPO0461	homoserine kinase	**1.411**	(0.03)		
	YPTB0623 *(carA)*	YPO0481	carbamoyl-phosphate synthase small chain	**0.609**	(< 0.001)	**0.747**	(0.028)
	YPTB0624 *(carB)*	YPO0482	carbamoyl-phosphate synthase large chain	**0.621**	(0.022)	**0.546**	(0.005)
	YPTB0676 *(ilvH)*	YPO0540	acetolactate synthase isozyme III small subunit	**1.68**	(0.006)		
	YPTB0711 *(aroP)*	YPO3421	aromatic amino acid transport protein	**1.68**	(0.006)		
	YPTB0761 *(cysH)*	YPO3370	phosphoadenosine phosphosulfate reductase (pseudogene. F/S)			**1.593**	(0.002)
	YPTB0789	YPO3343	probable extracellular solute-binding protein	**0.462**	(0.001)		
	YPTB0911 *(aroL)*	YPO3215	shikimate kinase II	**0.601**	(0.03)		
	YPTB0920 *(brnQ)*	YPO3202	branched-chain amino acid transport system II carrier protein	**1.481**	(0.033)		
	YPTB1108 *(glnH)*	YPO2615	putative amino acid-binding protein precursoR	**0.564**	(0.005)		
	YPTB1186	YPO1155	putative amino acid transporteR			**1.736**	(0.017)
	YPTB1240	YPO1200	putative amino acid permease			**0.637**	(0.025)
	YPTB1241	YPO1201	putative amino acid decarboxylase			**0.557**	(0.024)
	YPTB1346	YPO1315	putative hydrolase (pseudogene. stop)	**1.578**	(< 0.001)	**0.669**	(0.001)
	YPTB1352 *(sdaC)*	YPO1321	serine transporteR	**0.526**	(0.028)		
	YPTB1362 *(potG)*	YPO1332	putrescine transport ATP-binding protein	**0.728**	(0.035)		
	YPTB1375 *(artM)*	YPO1349	arginine transport system permease protein	**1.648**	(0.014)	**0.572**	(0.007)
	YPTB1384 *(poxB)*	YPO1358	pyruvate dehydrogenase [cytochrome]			**1.521**	(0.009)
	YPTB1411 *(ansB)*	YPO1386	putative L-asparaginase II precursoR			**1.869**	(0.013)
	YPTB1434 *(aspC)*	YPO1410	aspartate aminotransferase	**1.447**	(0.004)		
	YPTB1438 *(pepN)*	YPO1414	putative aminopeptidase N	**1.432**	(0.041)		
	YPTB1541 *(ysuJ)*	YPO1529	putative decarboxylase	**2.092**	(< 0.001)		
	YPTB1621 *(aroP)*	YPO1743	aromatic amino acid transport protein	**1.68**	(0.006)		
	YPTB1641 *(hpaF)*	YPO1765	5-carboxymethyl-2-hydroxymuconate delta-isomerase	**1.812**	(0.015)		
	YPTB1656 *(ptrB)*	YPO1780	oligopeptidase B	**1.695**	(0.006)		
	YPTB1889 *(lysA)*	or1363	possible diaminopimelate decarboxylase	**1.621**	(0.01)	**0.45**	(< 0.001)
	YPTB2001 *(prsA)*	YPO2013	ribose-phosphate pyrophosphokinase	**0.55**	(< 0.001)		
	YPTB2019	YPO2037	conserved hypothetical protein	**1.56**	(0.021)		
	YPTB2067	YPO2138	putative aminotransferase			**1.978**	(< 0.001)
	YPTB2105 *(oppA)*	YPO2182	periplasmic oligopeptide-binding protein precursoR			**0.466**	(< 0.001)
	YPTB2108 *(oppD)*	YPO2185	oligopeptide transport ATP-binding protein		(< 0.001)		
	YPTB2126 *(trpB)*	YPO2204	tryptophan synthase beta chain	**2.22**	(< 0.001)		
	YPTB2258 *(mppA)*	YPO2339	putative periplasmic murein peptide-binding protein	**1.376**	(0.039)		
	YPTB2262 *(tyrR)*	YPO2344	transcriptional regulatory protein	**0.486**	(< 0.001)		
	YPTB2295 *(gloA)*	YPO2381	lactoylglutathione lyase	**1.666**	(< 0.001)	**1.229**	(0.042)
	YPTB2437 *(pepT)*	YPO1631	peptidase T	**0.488**	(0.007)		
	YPTB2548 *(glnH)*	YPO2511	putative glutamine-binding periplasmic protein			**1.797**	(0.014)
	YPTB2549 *(glnP)*	YPO2512	putative glutamine transport system permease			**1.704**	(0.024)
	YPTB2550 *(glnQ)*	YPO2513	putative glutamine transport ATP-binding protein			**1.56**	(0.027)
	YPTB2632 *(aroC)*	YPO2751	chorismate synthase	**0.675**	(0.031)		
	YPTB2698	YPO2975	putative aminotransferase	**2.151**	(< 0.001)		
	YPTB2714 *(cysK)*	YPO2992	cysteine synthase A	**2.108**	(0.005)		
	YPTB2723	YPO3002	putative permease	**1.328**	(0.036)		
	YPTB2725	YPO3004	putative aminopeptidase (pseudogene. F/S)	**1.526**	(0.022)	**1.685**	(0.006)
	YPTB2784 *(gcvR)*	YPO3063	glycine cleavage system transcriptional repressoR	**2.302**	(<0.001)		
	YPTB2869 *(glyA)*	YPO2907	serine hydroxymethyltransferase			**1.783**	(0.001)
	YPTB2882 *(yfhB)*	YPO2924	putative membrane protein			**1.737**	(0.006)
	YPTB2909	YPO2699	conserved hypothetical protein	**1.513**	(0.017)		
	YPTB2942 *(ureC)*	YPO2667	urease alpha subunit	**0.333**	(<0.001)		
	YPTB2943 *(ureB)*	YPO2666	urease beta subunit	**0.2**	(<0.001)		
	YPTB2944 *(ureA)*	YPO2665	urease gamma subunit	**0.324**	(<0.001)		
	YPTB2961 *(proX)*	YPO2645	glycine betaine-binding periplasmic protein			**0.637**	(0.009)
	YPTB2986	YPO1061	conserved hypothetical protein			**0.551**	(0.031)
	YPTB3006 *(dapD)*	YPO1041	2.3.4.5-tetrahydropyridine-2-carboxylate N-succinyltransferase	**0.398**	(< 0.001)	**1.445**	(0.005)
	YPTB3181 *(gcsH)*	YPO0906	glycine cleavage system H protein	**0.377**	(< 0.001)		
	YPTB3182 *(gcvT)*	YPO0907	aminomethyltransferase	**0.526**	(0.022)		
	YPTB3189 *(serA)*	YPO0914	D-3-phosphoglycerate dehydrogenase	**1.582**	(0.04)	**0.517**	(0.005)
	YPTB3214 *(proC)*	YPO0942	putative pyrroline-5-carboxylate reductase			**0.647**	(0.038)
	YPTB3474	YPO0584	putative symporter protein	**0.61**	(0.007)		
	YPTB3570 *(aroQ)*	YPO3660	putative class II dehydroquinase			**0.721**	(0.036)
	YPTB3658 *(metA)*	YPO3727	homoserine O-succinyltransferase	**1.776**	(0.018)		
	YPTB3749 *(aroB)*	YPO0152	3-dehydroquinate synthase	**0.612**	(0.039)		
	YPTB3813 *(gdhA)*	YPO3971	NADP-specific glutamate dehydrogenase	**1.671**	(0.005)		
	YPTB3853 *(cysM)*	or2495	pyridoxal-phosphate dependent protein (pseudogene. partial)			**0.415**	(< 0.001)
	YPTB3957	YPO4111	putative periplasmic solute-binding protein	**1.59**	(0.006)		
**F: nucleotide transport and metabolism**							
	YPTB0250 *(udp)*	YPO3786	uridine phosphorylase	**0.486**	(0.005)		
	YPTB0519 *(nrdD)*	YPO3454	anaerobic ribonucleoside-triphosphate reductase	**0.614**	(0.024)		
	YPTB0584 *(deoD)*	YPO0440	purine nucleoside phosphorylase			**0.607**	(0.025)
	YPTB0623 *(carA)*	YPO0481	carbamoyl-phosphate synthase small chain	**0.609**	(< 0.001)	**0.747**	(0.028)
	YPTB0624 *(carB)*	YPO0482	carbamoyl-phosphate synthase large chain	**0.621**	(0.022)	**0.546**	(0.005)
	YPTB0754 *(pyrG)*	YPO3377	CTP synthase	**0.469**	(< 0.001)		
	YPTB0901 *(gpt)*	YPO3225	xanthine-guanine phosphoribosyltransferase	**0.68**	(0.035)		
	YPTB0991 *(apt)*	YPO3123	adenine phosphoribosyltransferase	**1.447**	(0.042)		
	YPTB1253 *(nrdB)*	YPO1213	ribonucleoside-diphosphate reductase 1 beta chain			**0.489**	(0.003)
	YPTB1254 *(nrdA)*	YPO1214	ribonucleoside-diphosphate reductase 1 alpha chain	**0.328**	(< 0.001)	**0.707**	(0.014)
	YPTB1439 *(pyrD)*	YPO1415	dihydroorotate dehydrogenase			**1.331**	(0.037)
	YPTB2001 *(prsA)*	YPO2013	ribose-phosphate pyrophosphokinase	**0.55**	(< 0.001)		
	YPTB2102 *(tdk)*	YPO2176	thymidine kinase	**0.75**	(0.034)		
	YPTB2706 *(nupC)*	YPO2983	nucleoside permease	**1.674**	(0.018)		
	YPTB2781 *(purC)*	YPO3059	phosphoribosylaminoimidazole-succinocarboxamide synthase (pseudogene. IS	**0.598**	(0.009)		
	YPTB2794 *(upp)*	YPO2827	uracil phosphoribosyltransferase	**0.515**	(0.002)		
	YPTB2796 *(purN)*	YPO2829	putative phosphoribosylglycinamide formyltransferase			**1.842**	(0.038)
	YPTB2803 *(ppx)*	YPO2837	putative exopolyphosphatase			**0.564**	(< 0.001)
	YPTB2956 *(nrdI)*	YPO2650	NrdI protein homologue	**2.842**	(< 0.001)		
	YPTB2957 *(nrdE)*	YPO2649	ribonucleoside-diphosphate reductase 2 alpha chain	**4.745**	(< 0.001)		
	YPTB2958 *(nrdF)*	YPO2648	ribonucleoside-diphosphate reductase 2 beta chain	**2.671**	(0.002)		
	YPTB3544	YPO3689	putative ribonuclease			**1.543**	(0.022)
	YPTB3854	YPO4019	putative phosphoribosyl transferase protein			**0.487**	(0.001)
**G: carbohydrate transport and metabolism**							
	YPTB0074 *(pfkA)*	YPO0078	6-phosphofructokinase	**1.593**	(0.017)		
	YPTB0087 *(glpF)*	YPO0091	glycerol uptake facilitator protein	**0.488**	(0.041)		
	YPTB0241 *(ugpC)*	YPO3793	sn-glycerol-3-phosphate transport. ATP-binding protein	**1.725**	(0.001)		
	YPTB0542	YPO0402	PTS system. IIB component			**0.565**	(0.023)
	YPTB0548	YPO0408	putative aldolase			**1.968**	(0.021)
	YPTB0550	YPO0410	putative ABC transporter permease protein	**0.573**	(0.033)		
	YPTB0569	YPO0424	putative pectinesterase	**0.268**	(< 0.001)		
	YPTB0583 *(deoB)*	YPO0439	phosphopentomutase	**1.651**	(0.03)		
	YPTB0782 *(dhaK)*	YPO3350	putative dihydroxyacetone kinase			**0.683**	(0.028)
	YPTB0799	YPO3332	putative sugar ABC transporter. permease protein	**1.787**	(0.018)	**1.912**	(0.01)
	YPTB0803 *(fucR)*	YPO3327	putative deoR-family regulatory protein	**0.435**	(< 0.001)	**1.521**	(0.007)
	YPTB0804 *(araD)*	YPO3326	L-ribulose-5-phosphate 4-epimerase	**0.537**	(< 0.001)		
	YPTB0874	or0625	probable sugar aldolase	**1.455**	(0.042)	**1.627**	(0.011)
	YPTB1079	YPO2586	conserved hypothetical protein	**0.559**	(0.025)		
	YPTB1080	YPO2587	conserved hypothetical protein	**0.644**	(0.026)		
	YPTB1119 *(nagB)*	YPO2627	putative glucosamine-6-phosphate isomerase	**0.654**	(0.027)		
	YPTB1140	YPO1106	conserved hypothetical protein	**0.705**	(0.034)		
	YPTB1166 *(gpmA)*	YPO1133	phosphoglycerate mutase 1	**3.178**	(< 0.001)		
	YPTB1290 *(bglA)*	YPO1254	6-phospho-beta-glucosidase	**1.802**	(0.008)		
	YPTB1327	YPO1295	putative ABC transport integral membrane subunit	**1.976**	(0.009)		
	YPTB1381	YPO1355	conserved hypothetical protein	**0.683**	(0.031)		
	YPTB1522 *(mglB)*	YPO1507	galactose-binding protein	**0.56**	(0.002)	**1.47**	(0.029)
	YPTB1581	YPO1572	putative sugar transporteR	**0.587**	(0.023)		
	YPTB1600 *(ybtX)*	YPO1915	putative signal transduceR	**1.766**	(0.003)		
	YPTB1632 *(manZ)*	YPO1756	PTS system. mannose-specific IID component	**3.084**	(< 0.001)		
	YPTB1633 *(manY)*	YPO1757	PTS system. mannose-specific IIC component	**1.991**	(0.005)		
	YPTB1634 *(manX)*	YPO1758	PTS system. mannose-specific IIAB component	**2.674**	(< 0.001)		
	YPTB1687	YPO1814	putative sugar ABC transporter. ATP-binding protein	**1.865**	(0.016)		
	YPTB1930	YPO1932	putative sugar transporteR	**1.963**	(0.002)		
	YPTB1975	YPO1982	putative dehydrogenase			**0.63**	(0.012)
	YPTB2047 *(pykA)*	YPO2064	pyruvate kinase II	**0.486**	(< 0.001)		
	YPTB2082	YPO2156	conserved hypothetical protein	**0.538**	(0.002)	**1.487**	(0.03)
	YPTB2083 *(gapA)*	YPO2157	glyceraldehyde 3-phosphate dehydrogenase A			**0.712**	(0.027)
	YPTB2147	YPO2225	conserved hypothetical protein			**1.297**	(0.041)
	YPTB2190 *(mlc)*	YPO2268	putative ROK family transcriptional regulatory protein	**1.417**	(0.037)		
	YPTB2205	or3894	ABC sugar/ribose transporter. permease subunit			**1.61**	(0.036)
	YPTB2306 *(pykF)*	YPO2393	pyruvate kinase I	**2.282**	(< 0.001)		
	YPTB2318 *(ppsA)*	YPO2409	phosphoenolpyruvate synthase	**0.463**	(0.008)		
	YPTB2356 *(kduI)*	YPO1725	4-deoxy-L-threo-5-hexosulose-uronate ketol-isomerase			**1.444**	(0.047)
	YPTB2360	YPO1721	putative sugar ABC transporter (permease)	**1.901**	(0.032)		
	YPTB2463 *(ptsG)*	YPO1608	PTS system. glucose-specific IIBC component	**4.033**	(< 0.001)	**1.607**	(0.002)
	YPTB2515	YPO2474	conserved hypothetical protein	**0.593**	(0.036)		
	YPTB2518	YPO2477	putative solute-binding protein	**0.62**	(0.029)		
	YPTB2535 *(rbsC)*	YPO2499	putative sugar transport system. permease protein	**1.7**	(0.013)		
	YPTB2715 *(ptsH)*	YPO2993	PTS system. phosphocarrier protein	**1.697**	(0.001)		
	YPTB2716 *(ptsI)*	YPO2994	PTS sytem. enzyme I component	**1.945**	(< 0.001)		
	YPTB2717 *(crr)*	YPO2995	PTS system. glucose-specific IIA component	**1.611**	(0.002)	**0.731**	(0.028)
	YPTB2962	YPO2644	conserved hypothetical protein (pseudogene. IS100)	**2.68**	(< 0.001)		
	YPTB3078	YPO0834	putative PTS transport protein			**1.742**	(0.025)
	YPTB3190 *(rpiA)*	YPO0915	ribose 5-phosphate isomerase A	**0.601**	(0.021)	**0.641**	(0.039)
	YPTB3195 *(fbaA)*	YPO0920	fructose-bisphosphate aldolase class II	**1.325**	(0.048)	**0.699**	(0.015)
	YPTB3196 *(pgk)*	YPO0921	phosphoglycerate kinase	**1.366**	(0.024)		
	YPTB3229	YPO0957	putative sugar transport system permease protein	**0.626**	(0.018)		
	YPTB3230 *(mglA)*	YPO0958	putative sugar transport ATP-binding protein	**0.624**	(0.005)		
	YPTB3262	YPO0988	putative membrane protein	**2.167**	(< 0.001)		
	YPTB3268	YPO0995	Sodium:galactoside symporter family protein			**1.764**	(0.014)
	YPTB3479 *(exuT)*	YPO0577	ExuT transport protein	**1.866**	(0.001)		
	YPTB3495	YPO3550	probable phosphosugar isomerase			**0.736**	(0.042)
	YPTB3536 *(treB)*	YPO3697	PTS system. trehalose-specific IIBC component	**0.34**	(< 0.001)	**1.832**	(0.015)
	YPTB3537 *(treC)*	YPO3696	putative trehalose-6-phosphate hydrolase	**0.279**	(< 0.001)		
	YPTB3609	YPO3620	putative carbohydrate transport protein			**1.459**	(0.02)
	YPTB3642 *(lamB)*	YPO3711	maltoporin	**0.419**	(< 0.001)		
	YPTB3779 *(glpR)*	YPO0120	glycerol-3-phosphate repressor protein			**1.398**	(0.043)
	YPTB3783 *(glgP)*	YPO3938	glycogen phosphorylase	**1.516**	(0.021)	**1.484**	(0.028)
	YPTB3787 *(glgB)*	YPO3942	1.4-alpha-glucan branching enzyme	**0.603**	(0.001)		
**H: coenzyme metabolism**							
	YPTB0014 *(mobA)*	or5120	molybdopterin-guanine dinucleotide biosynthesis protein A			**0.703**	(0.032)
	YPTB0056 *(kbl)*	YPO0059	2-amino-3-ketobutyrate coenzyme A ligase	**0.639**	(0.02)		
	YPTB0134 *(ilvG)*	YPO3901	acetolactate synthase isozyme II large subunit			**0.627**	(0.039)
	YPTB0182 *(hemX)*	YPO3851	putative uroporphyrin-III C-methyltransferase			**0.695**	(0.033)
	YPTB0264	YPO3769	conserved hypothetical protein			**0.695**	(0.01)
	YPTB0290 *(thiC)*	YPO3739	thiamine biosynthesis protein ThiC	**2.329**	(0.012)		
	YPTB0344	YPO0286	putative coproporphyrinogen III oxidase	**1.636**	(0.015)		
	YPTB0463 *(ispB)*	YPO3513	octaprenyl-diphosphate synthase	**0.731**	(0.03)	**0.614**	(0.002)
	YPTB0559	or0393	hypothetical protein	**1.723**	(0.007)		(< 0.001)
	YPTB0561	or0395	putative protein involved in molybdopterin biosynthesis	**1.802**	(0.004)		(< 0.001)
	YPTB0616 *(rpsT)*	YPO0474	30S ribosomal protein S20			**0.59**	(0.002)
	YPTB0664	or0477	hypothetical protein	**0.754**	(0.035)	**1.423**	(0.01)
	YPTB0731 *(folK)*	YPO3400	2-amino-4-hydroxy-6-hydroxymethyldihydropteridine pyrophosphokinase	**0.572**	(0.009)		
	YPTB0739 *(fhuC)*	YPO3392	ferrichrome transport ATP-binding protein FhuC	**1.932**	(0.046)		
	YPTB0758 *(ygcM)*	YPO3373	putative 6-pyruvoyl tetrahydrobiopterin synthase family protein	**0.556**	(0.002)	**0.629**	(0.011)
	YPTB0761 *(cysH)*	YPO3370	phosphoadenosine phosphosulfate reductase (pseudogene. F/S)			**1.593**	(0.002)
	YPTB0935 *(ribH)*	YPO3182	6.7-dimethyl-8-ribityllumazine synthase			**0.695**	(0.01)
	YPTB0940 *(ispA)*	YPO3176	geranyltranstransferase	**0.635**	(0.026)		
	YPTB1003 *(wbyH)*	YPO3111	putative exported protein			**0.727**	(0.039)
	YPTB1091 *(lipA)*	YPO2598	lipoic acid synthetase	**0.558**	(0.002)		
	YPTB1163 *(pnuC)*	YPO1128	intergral membrane NMN transport protein PnuC			**1.675**	(0.004)
	YPTB1181 *(bioA)*	YPO1150	adenosylmethionine-8-amino-7-oxononanoate aminotransferase	**3.377**	(< 0.001)	**1.592**	(0.038)
	YPTB1183 *(bioF)*	YPO1152	8-amino-7-oxononanoate synthase	**3.799**	(< 0.001)		
	YPTB1184 *(bioC)*	YPO1153	biotin synthesis protein BioC	**1.784**	(0.004)	**1.636**	(0.011)
	YPTB1185 *(bioD)*	YPO1154	dethiobiotin synthetase	**2.499**	(0.002)		
	YPTB1343	YPO1312	putative siderophore ABC transporter. ATP-binding subunit	**2.467**	(< 0.001)		
	YPTB1384 *(poxB)*	YPO1358	pyruvate dehydrogenase [cytochrome]			**1.521**	(0.009)
	YPTB1885	or1359	possible ThiF family				(< 0.001)
	YPTB1886	or1360	conserved hypothetical protein				(< 0.001)
	YPTB1888	or1362	conserved hypothetical protein	**1.886**	(0.001)	**0.314**	(< 0.001)
	YPTB2033	YPO2050	conserved hypothetical protein	**0.542**	(0.002)		
	YPTB2136 *(btuR)*	YPO2214	cob(I)alamin adenosyltransferase	**1.975**	(0.002)		
	YPTB2191	YPO2269	putative dethiobiotin synthetase	**0.3**	(< 0.001)		
	YPTB2304 *(ribE)*	YPO2391	riboflavin synthase alpha chain	**1.44**	(0.012)		
	YPTB2459	or3719	hypothetical	**0.379**	(< 0.001)		
	YPTB2561 *(menF)*	YPO2528	menaquinone-specific isochorismate synthase			**0.522**	(0.011)
	YPTB3574	YPO3657	putative sodium/panthothenate symporter			**0.634**	(0.01)
**I: lipid metabolism**							
	YPTB0416 *(psd)*	YPO0364	phosphatidylserine decarboxylase proenzyme			**1.846**	(0.024)
	YPTB0434 *(aidB)*	YPO0383	putative acyl-CoA dehydrogenase	**1.613**	(0.049)		
	YPTB0558	or0392	possible acyl-CoA dehydrogenase	**1.628**	(0.042)		(< 0.001)
	YPTB0674	YPO0537	putative AMP-binding enzyme-family protein	**0.616**	(0.047)		
	YPTB0883 *(yafH)*	YPO3244	probable acyl-CoA dehydrogenase	**2.292**	(< 0.001)		
	YPTB1355	YPO1324	putative permease			**0.499**	(< 0.001)
	YPTB1450 *(fabA)*	YPO1430	3-hydroxydecanoyl-[acyl-carrier-protein] dehydratase			**0.685**	(0.033)
	YPTB1480	YPO1462	putative acyl carrier protein			**1.669**	(0.048)
	YPTB2242 *(acpD)*	YPO2323	acyl carrier protein phosphodiesterase			**0.703**	(0.013)
	YPTB2470 *(acpP)*	YPO1600	acyl carrier protein			**0.661**	(0.002)
	YPTB2473 *(fabH)*	YPO1597	3-oxoacyl-[acyl-carrier-protein] synthase III	**0.757**	(0.047)	**0.708**	(0.017)
	YPTB2626 *(fabB)*	YPO2757	3-oxoacyl-[acyl-carrier-protein] synthase I			**0.605**	(0.009)
	YPTB2993 *(lpxD)*	YPO1054	UDP-3-o-[3-hydroxymyristoyl] glucosamine N-acyltransferase	**1.258**	(0.018)	**0.761**	(0.007)
	YPTB3849	YPO4014	putative membrane protein			**1.549**	(0.012)
	YPTB3856	YPO4021	hypothetical protein			**0.58**	(0.012)
**J: translation, ribosomal structure and biogenesis**							
	YPTB0034 *(trmH)*	YPO0037	tRNA (guanosine-2'-O-)-methyltransferase			**1.673**	(0.023)
	YPTB0041 *(rph)*	YPO0044	ribonuclease PH			**0.667**	(0.005)
	YPTB0276 *(tufA)*	or0197	elongation factor Tu			**0.624**	(0.001)
	YPTB0279 *(rplK)*	YPO3751	50S ribosomal protein L11	**0.703**	(0.027)	**0.682**	(0.018)
	YPTB0280 *(rplA)*	YPO3750	50S ribosomal protein L1	**0.681**	(0.027)	**0.689**	(0.031)
	YPTB0281 *(rplJ)*	YPO3749	50S ribosomal protein L10			**0.612**	(0.017)
	YPTB0282 *(rplL)*	YPO3748	50S ribosomal protein L7/L12			**0.58**	(0.014)
	YPTB0408 *(efp)*	YPO0354	elongation factor P	**1.7**	(0.018)		
	YPTB0438 *(rpsF)*	YPO3539	30S ribosomal protein S6	**0.597**	(0.018)	**0.501**	(0.003)
	YPTB0441 *(rplI)*	YPO3536	50S ribosomal protein L9			**0.476**	(< 0.001)
	YPTB0464 *(rplU)*	YPO3512	50S ribosomal protein L21			**0.539**	(0.006)
	YPTB0465 *(rpmA)*	YPO3511	50S ribosomal protein L27	**0.538**	(0.002)	**0.66**	(0.026)
	YPTB0480 *(infB)*	YPO3496	translation initiation factor IF2-2 (pseudogene. inframe deletion)	**0.598**	(< 0.001)	**0.661**	(< 0.001)
	YPTB0483 *(rpsO)*	YPO3493	30S ribosomal protein S15			**0.647**	(0.004)
	YPTB0484 *(pnp)*	YPO3490	polyribonucleotide nucleotidyltransferase	**0.642**	(0.006)		
	YPTB0529 *(valS)*	YPO3443	valyl-tRNA synthetase			**0.648**	(0.004)
	YPTB0575 *(prfC)*	YPO0430	peptide chain release factor 3	**0.536**	(0.014)	**0.611**	(0.046)
	YPTB0732 *(pcnB)*	YPO3399	poly(A) polymerase	**0.74**	(0.025)		
	YPTB0794 *(map)*	YPO3337	methionine aminopeptidase	**3.582**	(< 0.001)		
	YPTB0834 *(rpsP)*	YPO3295	30S ribosomal protein S16			**0.682**	(0.044)
	YPTB0835 *(rimM)*	YPO3294	16S rRNA processing protein	**0.628**	(0.003)	**0.68**	(0.011)
	YPTB0836 *(trmD)*	YPO3293	tRNA (guanine-N1)-methyltransferase			**0.596**	(0.004)
	YPTB0844 *(yfiA)*	YPO3279	putative sigma 54 modulation protein	**0.141**	(< 0.001)	**2.388**	(0.03)
	YPTB0846 *(rluD)*	YPO3277	ribosomal large subunit pseudouridine synthase d			**0.718**	(0.04)
	YPTB1058	or0769	conserved hypothetical protein			**1.626**	(0.021)
	YPTB1138	YPO1104	conserved hypothetical protein	**1.634**	(0.005)		
	YPTB1366	YPO1336	putative RNA methyltransferase			**0.588**	(0.018)
	YPTB1411 *(ansB)*	YPO1386	putative L-asparaginase II precursoR			**1.869**	(0.013)
	YPTB1417 *(rpsA)*	YPO1392	30S ribosomal protein S1			**0.427**	(< 0.001)
	YPTB1436 *(asnS)*	YPO1412	asparaginyl-tRNA synthetase	**1.628**	(0.022)		
	YPTB1953	YPO1955	putative acetyltransferase			**0.594**	(0.017)
	YPTB2005 *(prfA)*	YPO2017	peptide chain release factor 1	**0.665**	(0.008)	**0.733**	(0.035)
	YPTB2135	YPO2213	putative RNA pseudouridylate synthase-family protein	**0.691**	(0.032)		
	YPTB2150	YPO2228	translation initiation factor SUI1 family protein	**1.574**	(0.008)		
	YPTB2328	YPO2420	probable formyl transferase			**0.578**	(0.015)
	YPTB2336 *(pheT)*	YPO2428	phenylalanyl-tRNA synthetase beta chain	**1.646**	(0.003)		
	YPTB2337 *(pheS)*	YPO2429	phenylalanyl-tRNA synthetase alpha chain			**0.615**	(0.016)
	YPTB2339 *(rplT)*	or3807	50S ribosomal protein L20			**0.719**	(0.017)
	YPTB2618 *(truA)*	YPO2766	tRNA pseudouridine synthase A			**0.52**	(0.01)
	YPTB2861	YPO2898	putative SpoU-family rRNA methylase	**0.586**	(< 0.001)		
	YPTB3000 *(frr)*	YPO1047	ribosome recycling factoR			**0.609**	(0.026)
	YPTB3002 *(tsf)*	YPO1045	elongation factor Ts			**0.611**	(0.047)
	YPTB3003 *(rpsB)*	YPO1044	30S ribosomal protein S2			**0.464**	(< 0.001)
	YPTB3009	YPO1038	Conserved hypothetical protein			**0.761**	(0.039)
	YPTB3126	or3195	Possible bacteriophage protein	**1.616**	(0.029)		
	YPTB3507 *(rpsI)*	YPO3562	30S ribosomal protein S9	**0.723**	(0.037)	**0.582**	(0.001)
	YPTB3674 *(rpsD)*	YPO0233	30S ribosomal protein S4			**0.576**	(0.002)
	YPTB3675 *(rpsK)*	YPO0232	30S ribosomal protein S11			**0.594**	(0.017)
	YPTB3676 *(rpsM)*	YPO0231	30S ribosomal protein S13			**0.518**	(0.005)
	YPTB3679 *(rplO)*	YPO0228	50S ribosomal protein L15			**0.593**	(0.008)
	YPTB3682 *(rplR)*	YPO0225	50S ribosomal protein L18			**0.563**	(0.005)
	YPTB3684 *(rpsH)*	or2793	30S ribosomal protein S8	**0.638**	(0.042)	**0.619**	(0.032)
	YPTB3687 *(rplX)*	YPO0221	50S ribosomal protein L24			**0.574**	(0.005)
	YPTB3688 *(rplN)*	YPO0220	50S ribosomal protein L14			**0.634**	(0.015)
	YPTB3689 *(rpsQ)*	YPO0219	30S ribosomal protein S17			**0.58**	(0.016)
	YPTB3691 *(rplP)*	YPO0217	50S ribosomal protein L16			**0.511**	(< 0.001)
	YPTB3692 *(rpsC)*	YPO0216	30S ribosomal protein S3			**0.642**	(0.028)
	YPTB3693 *(rplV)*	YPO0215	50S ribosomal protein L22			**0.466**	(0.003)
	YPTB3694 *(rpsS)*	YPO0214	30S ribosomal protein S19			**0.612**	(0.013)
	YPTB3695 *(rplB)*	YPO0213	50S ribosomal protein l2	**0.555**	(< 0.001)	**0.596**	(< 0.001)
	YPTB3696 *(rplW)*	YPO0212	50S ribosomal protein L23			**0.509**	(0.001)
	YPTB3698 *(rplC)*	YPO0210	50S ribosomal protein L3			**0.465**	(0.001)
	YPTB3699 *(rpsJ)*	YPO0209	30S ribosomal protein S10	**0.654**	(0.049)	**0.579**	(0.014)
	YPTB3702 *(tufA,tufB)*	or2775	elongation factor EF-Tu			**0.672**	(0.003)
	YPTB3703 *(fusA)*	YPO0202	elongation factor G			**0.536**	(0.002)
	YPTB3946 *(rnpA)*	YPO4101	ribonuclease P protein	**0.58**	(0.005)		
**K: transcription**							
	YPTB0035 *(spoT)*	YPO0038	guanosine-3'.5'-bisbis(diphosphate) 3'-pyrophosphydrolase	**0.684**	(0.011)		
	YPTB0100 *(cytR)*	YPO0108	transcriptional repressoR	**0.572**	(0.014)		
	YPTB0167 *(rho)*	YPO3867	transcription termination factoR	**0.467**	(< 0.001)		
	YPTB0263 *(rfaH)*	YPO3770	putative regulatory protein			**0.552**	(0.001)
	YPTB0278 *(nusG)*	YPO3752	transcription antitermination protein			**0.57**	(0.001)
	YPTB0284 *(rpoC)*	YPO3746	DNA-directed RNA polymerase beta' chain			**0.556**	(0.019)
	YPTB0291 *(rsd)*	YPO3737	regulator of sigma D	**1.963**	(< 0.001)	**0.632**	(0.002)
	YPTB0333	YPO0276	putative LysR-family transcriptional regulatoR			**0.62**	(0.044)
	YPTB0387 *(rhaR)*	YPO0333	L-rhamnose operon transcriptional activatoR	**0.546**	(0.025)		
	YPTB0479 *(nusA)*	YPO3497	N utilization substance protein A	**0.653**	(0.006)	**0.595**	(0.001)
	YPTB0599 *(rob)*	YPO0456	putative right origin-binding protein			**0.588**	(0.012)
	YPTB0601 *(arcA)*	YPO0458	aerobic respiration control protein	**0.465**	(0.001)		
	YPTB0658 *(rapA)*	YPO0517	RNA polymerase associated helicase	**0.547**	(0.004)		
	YPTB0712 *(pdhR)*	YPO3420	pyruvate dehydrogenase complex repressoR	**0.808**	(0.004)	**0.695**	(< 0.001)
	YPTB0776 *(rpoS)*	YPO3355	RNA polymerase sigma factor RpoS			**0.559**	(0.001)
	YPTB0803 *(fucR)*	YPO3327	putative deoR-family regulatory protein	**0.435**	(< 0.001)	**1.521**	(0.007)
	YPTB0820	YPO3310	putative transcriptional regulatory protein	**0.712**	(0.022)		
	YPTB0857 *(emrR)*	YPO3266	MarR-family transcriptional regulatory protein	**0.662**	(0.029)		
	YPTB1088 *(cspE)*	YPO2595	putative cold shock protein	**0.579**	(0.005)		
	YPTB1258 *(rcsB)*	YPO1218	probable two component response regulator component B			**0.693**	(0.016)
	YPTB1332 *(psaE)*	YPO1301	putative regulatory protein	**1.986**	(0.001)	**0.474**	(< 0.001)
	YPTB1392 *(cspD)*	YPO1366	cold shock-like protein	**0.46**	(0.008)		
	YPTB1423 *(cspE)*	YPO1398	putative cold shock protein	**0.579**	(0.005)		
	YPTB1610 *(thuR)*	or1188	putative ThuR. regulatory protein for trehalosemaltose transp...			**1.403**	(0.025)
	YPTB1721	YPO1849	conserved hypothetical (pseudogene. F/S)	**1.366**	(0.025)		
	YPTB1967 *(hutC)*	YPO1973	putative GntR-family transcriptional regulatory protein	**0.739**	(0.018)		
	YPTB2048 *(hexR)*	YPO2065	hex regulon repressoR			**1.51**	(0.013)
	YPTB2072 *(fadR)*	YPO2144	fatty acid metabolism regulatory protein			**1.494**	(0.01)
	YPTB2177 *(araC)*	YPO2258	arabinose operon regulatory protein			**1.591**	(0.002)
	YPTB2190 *(mlc)*	YPO2268	putative ROK family transcriptional regulatory protein	**1.417**	(0.037)		
	YPTB2230 *(rstA)*	YPO2308	two-component regulatory system. response regulator protein	**0.658**	(0.024)		
	YPTB2262 *(tyrR)*	YPO2344	transcriptional regulatory protein	**0.486**	(< 0.001)		
	YPTB2288 *(rovA)*	YPO2374	MarR-family transcriptional regulatory protein			**0.415**	(< 0.001)
	YPTB2367 *(kdgR)*	YPO1714	IclR-family transcriptional regulatory protein			**0.75**	(0.029)
	YPTB2414 *(cspC)*	or3750	cold shock protein			**0.685**	(0.014)
	YPTB2418	YPO1651	AsnC-family transcriptional regulatory protein	**0.435**	(< 0.001)		
	YPTB2534	YPO2498	putative LacI-family transcriptional regulatory protein			**2.012**	(0.005)
	YPTB2737	YPO3017	putative rpiR-family transcriptional regulatory protein	**1.601**	(0.036)		
	YPTB2763 *(narP)*	YPO3041	nitrate/nitrite response regulator protein NarP			**0.715**	(0.043)
	YPTB2860	YPO2897	conserved hypothetical protein	**1.578**	(0.011)	**0.632**	(0.01)
	YPTB2865	YPO2903	putative RNA-binding protein			**0.652**	(0.035)
	YPTB2890 *(rnc)*	YPO2718	ribonuclease III			**0.673**	(0.013)
	YPTB2897 *(rpoE)*	YPO2711	RNA polymerase sigma E factoR			**1.827**	(< 0.001)
	YPTB2939 *(ureG)*	YPO2670	urease accessory protein	**0.293**	(< 0.001)		
	YPTB3017 *(gcvA)*	YPO1029	glycine cleavage system transcriptional activatoR	**1.634**	(0.049)		
	YPTB3490	YPO3545	lysR-family transcriptional regulatory protein	**1.503**	(0.036)	**1.518**	(0.032)
	YPTB3514	YPO3570	BolA-like protein	**1.315**	(0.035)		
	YPTB3538 *(rnk)*	YPO3695	regulator of nucleoside diphosphate kinase	**0.564**	(0.016)		
	YPTB3577 *(fiS)*	or2359	DNA-binding protein Fis	**0.611**	(0.034)	**0.58**	(0.02)
	YPTB3579	YPO3651	Transcriptional regulator (pseudogene. inframe deletion)			**0.675**	(0.017)
	YPTB3764 *(greB)*	YPO0136	transcription elongation factor			**0.69**	(0.004)
	YPTB3779 *(glpR)*	YPO0120	glycerol-3-phosphate repressor protein			**1.398**	(0.043)
	YPTB3798 *(gntR)*	YPO3955	gluconate utilization system Gnt-I transcriptional repressoR			**1.589**	(0.042)
	YPTB3847 *(uhpA)*	YPO4012	two-component system response regulatoR			**2.004**	(0.006)
	YPTB3887	YPO4034	putative AraC-family transcriptional regulatory protein			**1.495**	(0.04)
**L: DNA replication, recombination and repair**							
	YPTB0046 *(radC)*	YPO0049	putative DNA repair protein	**1.544**	(0.026)	**0.575**	(0.006)
	YPTB0261	or0185	cytoplasmic Dnase (function similar to TatD)	**0.569**	(< 0.001)		
	YPTB0292	YPO3736	conserved hypothetical protein			**1.453**	(0.017)
	YPTB0297 *(hupA)*	YPO3731	DNA-binding protein HU-alpha			**0.455**	(< 0.001)
	YPTB0302 *(or0218)*	or0218	putative transposase	**1.478**	(0.046)	**1.834**	(0.004)
	YPTB0439 *(priB)*	YPO3538	primosomal replication protein n			**0.557**	(0.045)
	YPTB0498	YPO3475	conserved hypothetical protein	**0.554**	(0.013)		
	YPTB0579	YPO0434	putative metalloenzyme	**0.64**	(0.023)		
	YPTB0658 *(rapA)*	YPO0517	RNA polymerase associated helicase	**0.547**	(0.004)		
	YPTB0913 *(rdgC)*	YPO3212	possible recombination associated protein RdgC	**1.82**	(0.047)		
	YPTB0941 *(xseB)*	YPO3175	exodeoxyribonuclease VII small subunit	**0.647**	(0.044)		
	YPTB0962 *(hupB)*	YPO3154	DNA-binding protein HU-beta	**1.674**	(< 0.001)	**0.727**	(0.015)
	YPTB0964 *(ybaV)*	YPO3152	putative exported protein	**0.625**	(0.02)		
	YPTB1418 *(ihfB)*	YPO1393	integration host factor beta-subunit			**0.46**	(0.003)
	YPTB1799	or1306	putative modification methylase	**1.94**	(0.026)		
	YPTB2040 *(ruvA)*	YPO2057	Holliday junction DNA helicase	**1.225**	(0.04)		
	YPTB2140 *(topA)*	YPO2218	DNA topoisomerase I	**1.654**	(0.012)	**0.548**	(0.004)
	YPTB2221 *(ogt)*	YPO2299	putative methylated-DNA – protein-cysteine methyltransferase	**0.611**	(0.017)		
	YPTB2335 *(ihfA)*	YPO2427	integration host factor alpha-subunit	**1.626**	(0.001)	**0.623**	(0.001)
	YPTB2458	or3720	hypothetical	**0.501**	(< 0.001)		
	YPTB2792	YPO3071	conserved hypothetical protein			**1.337**	(0.026)
	YPTB2834 *(xseA)*	YPO2872	exodeoxyribonuclease VII large subunit			**0.639**	(0.01)
	YPTB3389	YPO0674	putative MutT-family protein	**1.299**	(0.044)		
	YPTB3577 *(fiS)*	or2359	DNA-binding protein Fis	**0.611**	(0.034)	**0.58**	(0.02)
	YPTB3757	YPO0144	putative hydrolase	**1.58**	(0.013)		
**M: cell envelope biogenesis, outer membrane**							
	YPTB0051 *(kdtX)*	YPO0054	lipopolysaccharide core biosynthesis glycosyl transferase	**0.592**	(0.001)		
	YPTB0173 *(rffH)*	YPO3861	glucose-1-phosphate thymidylyltransferase			**1.604**	(0.028)
	YPTB0415	YPO0363	putative membrane transport protein			**1.853**	(0.003)
	YPTB0491	YPO3483	multidrug efflux protein			**1.833**	(0.032)
	YPTB0493 *(ibeB)*	YPO3481	probable outer membrane efflux lipoprotein	**1.863**	(0.015)		
	YPTB0694 *(lpxC)*	YPO0561	UDP-3-O-[3-hydroxymyristoyl] N-acetylglucosamine deacetylase	**0.787**	(0.04)		
	YPTB0775 *(nlpD)*	YPO3356	lipoprotein	**1.345**	(0.019)	**0.732**	(0.015)
	YPTB0906	YPO3220	conserved hypothetical protein			**0.651**	(0.038)
	YPTB0955 *(yajG)*	YPO3161	putative lipoprotein	**1.48**	(0.021)		
	YPTB0987 *(kefA)*	YPO3129	putative potassium efflux system	**0.626**	(0.001)		
	YPTB1002 *(prt)*	YPO3112	paratose synthase			**0.706**	(0.013)
	YPTB1008 *(wbyK)*	YPO3104	putative mannosyltransferase	**0.527**	(0.001)	**0.669**	(0.03)
	YPTB1014 *(wzz)*	YPO3096	O-antigen chain length determinant			**0.577**	(0.019)
	YPTB1109 *(cutE)*	YPO2616	putative apolipoprotein N-acyltransferase	**0.669**	(0.029)		
	YPTB1160 *(pal)*	YPO1125	peptidoglycan-associated lipoprotein Pal	**1.649**	(< 0.001)	**0.553**	(< 0.001)
	YPTB1217 *(pbpG)*	YPO1176	penicillin-binding protein 7 precursoR	**0.565**	(0.009)		
	YPTB1261 *(ompC)*	YPO1222	outer membrane protein C. porin	**0.284**	(< 0.001)	**1.371**	(0.034)
	YPTB1266 *(pla2)*	YPO1231	putative outer membrane-associated protease	**0.569**	(0.015)		
	YPTB1309 *(spr)*	YPO1275	putative lipoprotein	**0.499**	(< 0.001)	**0.646**	(0.002)
	YPTB1381	YPO1355	conserved hypothetical protein	**0.683**	(0.031)		
	YPTB1435	YPO1411	putative outer membrane porin C protein				(< 0.001)
	YPTB1453 *(ompA)*	YPO1435	putative outer membrane porin A protein			**0.355**	(< 0.001)
	YPTB1514	YPO1498	putative exported protein			**0.301**	(< 0.001)
	YPTB1528 *(yohK)*	YPO1513	putative membrane protein	**1.513**	(0.043)		
	YPTB1731	YPO1860	attachment invasion locus protein			**1.634**	(< 0.001)
	YPTB1819	or1328	hypothetical phage protein	**1.902**	(0.025)		
	YPTB1964	or1419	putative outer membrane porin C protein				(< 0.001)
	YPTB1975	YPO1982	putative dehydrogenase			**0.63**	(0.012)
	YPTB2081	YPO2155	putative exported protein	**0.796**	(0.009)		
	YPTB2113	YPO2190	attachment invasion locus protein precursoR	**1.511**	(0.012)		(< 0.001)
	YPTB2117 *(tonB)*	YPO2193	TonB	**5.465**	(< 0.001)		
	YPTB2123 *(ompW)*	YPO2201	putative exported protein	**0.165**	(< 0.001)		
	YPTB2233 *(sepC)*	YPO2312	insecticidal toxin (pseudogene. inframe insertion)	**1.424**	(0.045)		
	YPTB2294 *(sepC)*	YPO2380	insecticidal toxin (pseudogene. inframe insertion)	**1.424**	(0.045)		
	YPTB2323 *(nlpC)*	YPO2415	putative lipoprotein	**1.388**	(0.025)		
	YPTB2979 *(cutF)*	YPO1067	putative copper homeostasis lipoprotein	**1.794**	(0.001)		
	YPTB2994 *(ompH)*	YPO1053	cationic 19 kDa outer membrane protein precursoR	**1.69**	(< 0.001)	**0.538**	(< 0.001)
	YPTB2995	YPO1052	putative surface antigen			**0.745**	(0.016)
	YPTB2996	YPO1051	putative membrane protein	**1.531**	(0.03)		
	YPTB3194	YPO0919	putative membrane protein	**1.569**	(0.024)		
	YPTB3277	or3091	Conserved hypothetical protein			**1.57**	(0.026)
	YPTB3282	or3086	Conserved hypothetical protein (partial. c-term)	**1.529**	(0.025)		
	YPTB3285	or3082	Putative autotransporter secreted protein			**1.593**	(0.028)
	YPTB3313 *(slyB)*	YPO0752	putative lipoprotein	**1.452**	(0.003)		
	YPTB3407 *(rfaE)*	YPO0654	ADP-heptose synthase	**0.709**	(0.042)		
	YPTB3438	YPO0617	putative membrane protein	**1.993**	(0.004)		
	YPTB3497 *(mtgA)*	YPO3552	monofunctional biosynthetic peptidoglycan transglycosylase			**1.736**	(0.008)
	YPTB3513 *(murA)*	YPO3569	UDP-N-acetylglucosamine1-carboxyvinyltransferase			**0.775**	(0.034)
	YPTB3717	YPO0187	putative glycosyl transferase	**0.657**	(0.013)		
	YPTB3958	YPO4112	putative membrane protein	**1.69**	(0.004)		
	YPTB3965 *(glmU)*	YPO4119	UDP-N-acetylglucosamine pyrophosphorylase	**1.572**	(0.042)		
**N: cell motility and secretion**							
	YPTB0071 *(cpxP)*	YPO0075	putative exported protein			**0.337**	(< 0.001)
	YPTB0156	YPO3881	putative chaperone protein	**0.657**	(0.01)		
	YPTB0158	YPO3879	putative outer membrane usher protein			**1.626**	(0.016)
	YPTB0359	YPO0302	putative outer membrane fimbrial usher protein	**1.398**	(0.049)		
	YPTB0706 *(hofB)*	YPO3426	putative type II secretion system protein	**0.711**	(0.043)		
	YPTB1335 *(psaB)*	YPO1304	chaperone protein PsaB precursoR	**0.216**	(< 0.001)		
	YPTB1680 *(flgJ)*	YPO1807	flagellar protein FlgJ			**1.906**	(0.01)
	YPTB1681 *(flgK)*	YPO1808	flagellar hook-associated protein 1	**0.616**	(0.01)	**0.682**	(0.034)
	YPTB1682 *(flgL)*	YPO1809	flagellar hook-associated protein 3	**1.514**	(0.041)		
	YPTB1693	YPO1820A				**2.053**	(0.011)
	YPTB1695 *(fliN)*	YPO1822	flagellar motor switch protein FliN			**1.79**	(0.021)
	YPTB1698 *(fliK)*	YPO1825	flagellar hook-length control protein FliK			**1.941**	(0.006)
	YPTB1919	YPO1920	probable fimbrial usher protein			**1.461**	(0.019)
	YPTB2396 *(cheZ)*	YPO1681	chemotaxis protein CheZ			**1.477**	(0.027)
	YPTB2405 *(cheA)*	YPO1666	chemotaxis protein CheA	**1.725**	(0.015)		
	YPTB2843	YPO2881	putative fimbrial biogenesis protein			**0.686**	(0.022)
	YPTB3347 *(fliG)*	YPO0715	puative flagellar motor switch protein	**1.68**	(0.03)	**1.722**	(0.024)
	YPTB3357	YPO0704	flagellar assembly protein	**0.611**	(0.049)		
	YPTB3896	YPTB3896	fimbrial protein			**1.644**	(0.021)
**No COG**							
	YPTB0092	YPO0100	hypothetical protein	**1.623**	(0.025)	**0.434**	(< 0.001)
	YPTB0094	YPO0102	putative exported protein			**0.503**	(< 0.001)
	YPTB0123 *(yijD)*	YPO3912	putative membrane protein			**0.669**	(0.033)
	YPTB0139	YPO3895	putative membrane protein			**0.717**	(0.036)
	YPTB0141	YPTB0141	putative membrane protein	**1.774**	(0.026)		
	YPTB0148	or0096	colicin (pseudogene. partial)	**1.493**	(0.013)		
	YPTB0149	or0097	putative colicin immunity protein			**1.605**	(0.045)
	YPTB0151 *(imm2)*	or0099	pyocin S2 immunity protein	**1.549**	(0.013)	**0.486**	(< 0.001)
	YPTB0212 *(dcrB)*	YPO3823	putative lipoprotein	**0.758**	(0.028)		
	YPTB0237	or5000	putative exported protein			**1.357**	(0.048)
	YPTB0244	or0169	hypothetical	**0.517**	(< 0.001)		
	YPTB0362	YPO0306	conserved hypothetical protein (pseudogene. F/S)			**0.66**	(0.036)
	YPTB0391	YPO0337	putative exported protein			**0.527**	(< 0.001)
	YPTB0406	YPO0352	putative lipoprotein	**1.294**	(0.042)		
	YPTB0449	YPO3527	conserved hypothetical protein			**2**	(< 0.001)
	YPTB0499	YPO3474	hypothetical protein			**1.509**	(0.036)
	YPTB0505	YPO3468	hypothetical protein			**1.632**	(0.026)
	YPTB0546	YPO0406	putative exported protein	**2.397**	(< 0.001)		
	YPTB0560	or0394	hypothetical protein	**1.636**	(0.028)	**0.271**	(< 0.001)
	YPTB0593	YPO0450	putative membrane protein	**0.736**	(0.025)	**1.464**	(0.007)
	YPTB0651	YPO0511	hypothetical protein			**0.576**	(0.02)
	YPTB0657	YPO0516	hypothetical protein			**0.457**	(0.001)
	YPTB0666	or4788	putative IS1400 transposase B	**2.101**	(< 0.001)		
	YPTB0678	YPO0544	putative membrane protein			**0.631**	(0.009)
	YPTB0768 *(ygbE)*	YPO3363	putative membrane protein	**1.735**	(0.007)		
	YPTB0793	YPO3339	hypothetical protein	**1.679**	(0.023)	**1.94**	(0.005)
	YPTB0795	YPO3336	conserved hypothetical protein	**3**	(< 0.001)		
	YPTB0903 *(crl)*	YPO3223	curlin genes regulatory protein	**2.098**	(< 0.001)		
	YPTB0957	YPO3159	hypothetical protein			**1.521**	(0.034)
	YPTB0978 *(ymoA)*	YPO3138	modulating protein YmoA (histone-like protein)	**0.509**	(< 0.001)		
	YPTB0979	YPO3137	conserved hypothetical protein	**0.469**	(< 0.001)		
	YPTB0980	YPO3136	hypothetical protein	**0.587**	(0.01)		
	YPTB1004 *(wzx)*	or0734	putative O-unit flippase			**0.642**	(0.007)
	YPTB1018 *(ushB)*	or0747	5'-nucleotidase/UDP-sugar diphosphatase			**1.34**	(0.031)
	YPTB1041	YPO2820	hypothetical protein	**1.394**	(0.029)		
	YPTB1042 *(int)*	or4598	phage integrase (pseudogene. Partial)			**1.483**	(0.046)
	YPTB1043	or0754	hypothetical	**0.569**	(0.002)		
	YPTB1130 *(trp1400A)*	or4531	IS1400 transposase A			**2.136**	(< 0.001)
	YPTB1167 *(psiF)*	YPO1134	putative starvation-inducible protein	**2.04**	(0.014)		
	YPTB1202 *(xapB)*	YPO1172	xanthosine permease (pseudogene. IS1541)	**1.381**	(0.03)	**1.499**	(0.009)
	YPTB1220	YPO1179	conserved hypothetical protein	**0.702**	(0.038)		
	YPTB1287	or0929	putative bacteriophage tail fiber protein	**1.853**	(0.01)		
	YPTB1291	YPO1255	hypothetical protein	**0.361**	(< 0.001)		
	YPTB1303	YPO1269	conserved hypothetical protein	**1.754**	(0.008)		
	YPTB1334 *(psaA)*	YPO1303	pH 6 antigen precursor (antigen 4) (adhesin)		(< 0.001)		
	YPTB1359	YPO1328	putative membrane protein	**1.723**	(0.003)		
	YPTB1515	YPO1499	putative membrane protein	**1.521**	(0.045)		
	YPTB1543 *(ysuH)*	YPO1531	putative siderophore biosynthetic enzyme	**1.896**	(0.02)		
	YPTB1583	YPO1574	putative exported protein	**0.605**	(< 0.001)		
	YPTB1602 *(int)*	or4274	integrase	**0.592**	(0.026)		
	YPTB1616	or1193	conserved hypothetical protein	**0.677**	(0.016)	**1.488**	(0.014)
	YPTB1619	YPO1741	hypothetical protein			**1.496**	(0.025)
	YPTB1622	YPO1744	putative exported protein	**1.671**	(0.019)		
	YPTB1663	YPO1788	putative acyl carrier protein	**1.57**	(0.049)		
	YPTB1664	YPO1789	putative membrane protein	**1.638**	(0.031)		
	YPTB1668 *(invA)*	or1234	putative invasin	**0.5**	(< 0.001)	**0.397**	(< 0.001)
	YPTB1705	YPTB1705	putative phage minor tail protein	**1.794**	(0.002)		
	YPTB1722	YPO1850	hypothetical protein			**1.593**	(0.045)
	YPTB1734	YPO1864	conserved hypothetical protein	**0.679**	(0.003)		
	YPTB1752	or4178	O protein [Enterobacteria phage 186] gb|AAC34159.1| (U32222)...	**0.49**	(0.01)		
	YPTB1785	or4145	hypothetical protein	**0.453**	(< 0.001)		
	YPTB1786	or4144	hypothetical protein			**1.605**	(0.042)
	YPTB1798 *(yfdM)*	or1305	conserved hypothetical protein	**1.506**	(0.043)		
	YPTB1801	or1309	hypothetical protein	**0.279**	(< 0.001)		
	YPTB1802	or1310	hypothetical protein	**0.449**	(0.01)		
	YPTB1815	or1324	putative phage protein	**1.57**	(0.037)		
	YPTB1821	YPTB1821	putative acyl carrier protein	**1.57**	(0.049)		
	YPTB1822	or4135	putative membrane protein	**1.638**	(0.031)		
	YPTB1826	or1333	bacteriophage hypothetical protein			**1.754**	(0.04)
	YPTB1850	or4114	gpR [Enterobacteria phage P2] sp|P36933|VPR_BPP2 tAIL COMPLE...			**1.779**	(0.024)
	YPTB1858	or4106	similar to V protein phage 186			**1.743**	(0.024)
	YPTB1862	or4102	putative phage replication protein	**0.536**	(0.011)		
	YPTB1884	or1358	possible MFS Superfamliy multidrug-efflux transporter				(< 0.001)
	YPTB1887	or1361	hypothetical protein				(< 0.001)
	YPTB1893	YPO1874	conserved hypothetical protein	**1.593**	(0.034)		
	YPTB1980	YPO1987	hypothetical protein (pseudogene. IS285)			**1.485**	(0.047)
	YPTB1986	YPO1994	hypothetical protein	**0.649**	(0.039)		
	YPTB1987	YPO1995	hypothetical protein	**0.57**	(0.047)		
	YPTB1996	YPO2004	putative membrane protein			**2.041**	(0.002)
	YPTB2000	YPO2012	putative membrane protein	**0.256**	(< 0.001)		
	YPTB2092	YPO2166	putative exported protein	**0.671**	(0.027)		
	YPTB2114	YPO2191	hypothetical protein	**0.28**	(< 0.001)		
	YPTB2148	YPO2226	hypothetical protein	**1.855**	(0.007)		
	YPTB2151 *(osmB)*	YPO2229	osmotically inducible lipoprotein B precursoR	**2.229**	(< 0.001)	**1.413**	(0.032)
	YPTB2219	YPO2297	hypothetical protein			**1.576**	(0.031)
	YPTB2227	YPO2305	putative exported protein	**1.37**	(0.015)	**0.774**	(0.042)
	YPTB2229	YPO2307	conserved hypothetical protein	**0.708**	(0.026)		
	YPTB2237 *(asr)*	YPO2318	putative acid shock protein			**0.654**	(0.022)
	YPTB2269 *(pspB)*	YPO2350	phage shock protein B	**1.983**	(0.018)		
	YPTB2334	YPO2426	putative exported protein	**1.628**	(0.015)		
	YPTB2353 *(yfeE)*	YPO2445	putative yfeABCD locus regulatoR	**1.526**	(0.008)		
	YPTB2358 *(pelY)*	YPO1723	periplasmic pectate lyase precursoR			**2.081**	(0.001)
	YPTB2363	YPO1718	putative exported protein			**1.804**	(0.038)
	YPTB2387	YPO1694	conserved hypothetical protein	**1.794**	(0.008)		
	YPTB2393	YPO1686	putative exported protein			**1.442**	(0.019)
	YPTB2417 *(ylaC)*	YPO1652	putative membrane protein			**1.559**	(0.003)
	YPTB2419	YPO1650	hypothetical protein	**0.6**	(0.026)		
	YPTB2420	YPO1649	conserved hypothetical protein	**1.758**	(0.004)	**2.658**	(< 0.001)
	YPTB2421	YPO1648	probable histidine acid phosphatase			**1.474**	(0.045)
	YPTB2425	YPO1643	hypothetical protein	**1.662**	(0.01)		
	YPTB2446	YPO1619	hypothetical protein	**1.504**	(0.019)		
	YPTB2483 *(dinI)*	YPO1586	DNA-damage-inducible protein I	**1.646**	(0.006)		
	YPTB2495	or1732	glucans biosynthesis protein (pseudogene. deletions)	**1.503**	(0.024)		
	YPTB2496	or1737	hypothetical			**0.433**	(0.001)
	YPTB2540	or3654	conserved hypothetical protein			**2.145**	(0.008)
	YPTB2552	YPO2515	hypothetical	**1.46**	(0.023)	**1.396**	(0.042)
	YPTB2554	YPO2521	putative exported protein	**0.648**	(0.044)		
	YPTB2562	YPO2530	conserved hypothetical protein			**0.527**	(0.026)
	YPTB2623 *(flk)*	YPO2760	putative flagellar assembly regulatory protein. flk	**0.549**	(0.008)		
	YPTB2699	YPO2976	conserved hypothetical protein	**4.448**	(< 0.001)	**1.458**	(0.016)
	YPTB2704	YPO2981	putative exported protein	**1.628**	(0.015)		
	YPTB2744 *(yfeY)*	YPO3026	putative lipoprotein			**1.604**	(0.019)
	YPTB2753	YPO3031	putative acetyltransferase	**1.391**	(0.02)		
	YPTB2750	or3477	hypothetical			**0.688**	(0.006)
	YPTB2787	YPO3066	hypothetical protein	**1.504**	(0.019)		
	YPTB2822	YPO2857	putative exported protein			**1.718**	(0.031)
	YPTB2877	YPO2918	putative exported protein	**0.542**	(0.024)		
	YPTB2893	YPO2715	putative membrane protein	**1.565**	(0.011)		
	YPTB2922	or3339	hypothetical	**1.886**	(0.001)		
	YPTB2935	YPO2674	putative exported protein		(< 0.001)		
	YPTB2951	YPO2657	putative mobilization protein	**0.566**	(< 0.001)		
	YPTB2953	YPO2653	conserved hypothetical protein			**0.639**	(0.027)
	YPTB2954 *(asr)*	YPO2652	putative acid shock protein	**1.815**	(0.033)		
	YPTB3007	YPO1040	conserved hypothetical protein	**0.728**	(0.024)	**0.73**	(0.025)
	YPTB3039	YPO0791	hypothetical protein	**0.545**	(0.007)		
	YPTB3041 *(ygeD)*	YPO0792	putative membrane protein			**0.617**	(0.015)
	YPTB3071	YPO0822	putative exported protein	**0.51**	(0.002)		
	YPTB3111	YPO0867	putative membrane protein	**0.675**	(0.027)		
	YPTB3177	YPO0901	putative exported protein			**1.442**	(0.019)
	YPTB3179	YPO0904	hypothetical protein			**1.708**	(0.002)
	YPTB3220	YPO0948	conserved hypothetical protein	**1.56**	(0.021)		
	YPTB3256 *(insA)*	or3105	insertion element protein			**1.939**	(< 0.001)
	YPTB3257	YPO0983	putative lipoprotein			**2.06**	(0.001)
	YPTB3280	or3088	Hypothetical	**0.606**	(0.009)		
	YPTB3305	YPO1002	hypothetical protein	**1.679**	(0.023)	**1.94**	(0.005)
	YPTB3342	YPO0720	putative flagellar regulatory protein			**1.492**	(0.007)
	YPTB3343	YPO0719	hypothetical protein			**1.563**	(0.038)
	YPTB3371	YPO0694	Putative membrane protein (pseudogene. inframe deletion)	**0.653**	(0.049)		
	YPTB3421	YPO0640	hypothetical protein	**0.716**	(0.028)		
	YPTB3454	or2954	Fragment of hemagglutinin/hemolysin-related protein	**0.653**	(0.025)		
	YPTB3458	or2950	hypothetical			**1.92**	(0.005)
	YPTB3504	YPO3559	putative exported protein	**0.483**	(< 0.001)		
	YPTB3534	YPO3699	putative exported protein			**0.676**	(0.038)
	YPTB3551	YPO3681	Insecticidal toxin TcaA			**0.619**	(0.047)
	YPTB3556	YPO3675	putative exported protein			**0.637**	(0.043)
	YPTB3627	YPO3601	conserved hypothetical protein	**0.595**	(0.006)		
	YPTB3641 *(malM)*	YPO3710	maltose operon periplasmic protein	**0.379**	(< 0.001)		
	YPTB3769 *(feoC)*	YPO0131	ferrous iron transport protein C	**1.899**	(< 0.001)		
	YPTB3770	YPO0130	putative exported protein			**0.43**	(< 0.001)
	YPTB3781	YPO3935	putative membrane protein			**0.717**	(0.036)
	YPTB3789	or2712	putative invasin			**0.614**	(0.01)
	YPTB3811 *(uspB)*	YPO3969	universal stress protein B			**0.665**	(0.04)
	YPTB3834 *(pelY)*	YPO3994	periplasmic pectate lyase precursoR			**2.081**	(0.001)
	YPTB3835	YPO3995	putative exported protein			**1.804**	(0.038)
	YPTB3855	YPO4020	putative membrane protein			**0.422**	(< 0.001)
	YPTB3893	YPO4040	putative exported protein			**0.487**	(< 0.001)
	YPTB3908	YPO4081	putative membrane protein			**1.408**	(0.01)
	YPTB3917 *(yiaF)*	YPO4070	putative exported protein	**1.554**	(0.016)	**0.701**	(0.047)
	YPTB3922	YPO4064	hypothetical protein			**1.409**	(0.046)
	YPTB3923	YPO4063	putative membrane protein			**1.53**	(0.041)
	YPTB3944	or2545	hypothetical protein_	**0.593**	(0.016)		
**O: posttranslational modification, protein turnover, chaperones**							
	YPTB0404 *(groES)*	YPO0350	10 kDa chaperonin	**0.683**	(0.029)	**1.445**	(0.035)
	YPTB0427 *(hflK)*	YPO0375	putative membrane protein (pseudogene. inframe deletion)			**0.619**	(0.033)
	YPTB0494	YPO3480	conserved hypothetical protein	**0.444**	(< 0.001)		
	YPTB0495	YPO3479	putative protease	**0.328**	(< 0.001)		
	YPTB0518 *(nrdG)*	YPO3455	anaerobic ribonucleoside-triphosphate reductase Activating protein	**0.439**	(0.001)		
	YPTB0612 *(dnaK)*	YPO0469	chaperone protein DnaK	**0.73**	(0.049)	**0.693**	(0.025)
	YPTB0647 *(clpB)*	YPO0506	putative Clp ATPase	**1.481**	(0.047)	**0.402**	(< 0.001)
	YPTB0774 *(pcm)*	YPO3357	protein-L-isoaspartate O-methyltransferase	**0.62**	(0.014)		
	YPTB0925 *(ahpC)*	YPO3194	putative alkyl hydroperoxide reductase subunit c			**0.545**	(< 0.001)
	YPTB0948 *(cyoE)*	YPO3168	protoheme IX farnesyltransferase	**1.422**	(0.039)		
	YPTB0958 *(tig)*	YPO3158	Trigger factoR			**0.607**	(0.008)
	YPTB0995 *(htpG)*	YPO3119	heat shock protein HtpG			**0.569**	(0.004)
	YPTB1025	YPO3083	conserved hypothetical protein			**0.66**	(0.014)
	YPTB1026 *(ybbN)*	YPO3082	putative thioredoxin			**0.715**	(0.019)
	YPTB1034 *(ppiB)*	YPO3074	peptidyl-prolyl cis-trans isomerase B	**1.419**	(0.013)		
	YPTB1141	YPO1107	heat shock protein GrpE	**1.442**	(0.048)		
	YPTB1406 *(pflA)*	YPO1381	pyruvate formate-lyase 1 activating enzyme	**0.578**	(0.004)		
	YPTB1871	or1348	similar to hypothetical bacteriophage P27 protein	**0.58**	(0.001)		
	YPTB1945	YPO1947	putative thioredoxin	**1.646**	(0.002)		
	YPTB2070 *(dsbB)*	YPO2141	disulfide bond formation protein B	**1.636**	(< 0.001)	**0.757**	(0.023)
	YPTB2084	YPO2158	conserved hypothetical protein	**0.683**	(0.003)		
	YPTB2261 *(tpx)*	YPO2342	thiol peroxidase	**2.414**	(< 0.001)	**0.527**	(< 0.001)
	YPTB2297	YPO2383	conserved hypothetical protein			**0.741**	(0.025)
	YPTB2311	YPO2401	conserved hypothetical protein	**1.822**	(0.001)		
	YPTB2312	YPO2402	putative ATP-dependent transporteR	**2.036**	(0.006)		
	YPTB2313	YPO2403	conserved hypothetical protein	**2.213**	(0.002)		
	YPTB2734 *(cysT)*	YPO3014	sulfate transport system permease protein CysT			**0.714**	(0.029)
	YPTB2785 *(bcp)*	YPO3064	bacterioferritin comigratory protein	**1.417**	(0.033)		
	YPTB2806	YPO2840	putative heat shock protein	**0.674**	(0.027)		
	YPTB2905 *(pcp)*	YPO2703	putative pyrrolidone-carboxylate peptidase	**1.608**	(0.016)		
	YPTB2938 *(ureD)*	YPO2671	urease accessory protein	**0.377**	(< 0.001)		
	YPTB2939 *(ureG)*	YPO2670	urease accessory protein	**0.293**	(< 0.001)		
	YPTB2940 *(ureF)*	YPO2669	urease accessory protein	**0.268**	(< 0.001)		
	YPTB2941 *(ureE)*	YPO2668	urease accessory protein	**0.347**	(< 0.001)		
	YPTB3408 *(glnE)*	YPO0653	glutamate-ammonia-ligase adenylyltransferase	**1.792**	(0.046)		
	YPTB3415 *(gcp)*	YPO0646	putative glycoprotease	**0.568**	(0.009)		
	YPTB3710 *(fkpA)*	YPO0195	peptidyl-prolyl cis-trans isomerase			**0.571**	(0.007)
	YPTB3728	YPO0176	conserved hypothetical protein	**1.988**	(0.002)		
	YPTB3734 *(ppiA)*	YPO0167	peptidyl-prolyl cis-trans isomerase A	**0.757**	(0.015)	**0.61**	(< 0.001)
	YPTB3930 *(fdhE)*	YPO4055	putative formate dehydrogenase formation protein	**0.556**	(< 0.001)	**0.798**	(0.031)
**P: inorganic ion transport and metabolism**							
	YPTB0071 *(cpxP)*	YPO0075	putative exported protein			**0.337**	(< 0.001)
	YPTB0270 *(trkH)*	YPO3762	Trk system potassium uptake protein TrkH	**0.634**	(0.018)		
	YPTB0336 *(hmuV)*	YPO0279	hemin transport system ATP-binding protein	**1.666**	(0.003)	**0.652**	(0.01)
	YPTB0338 *(hmuT)*	YPO0281	hemin-binding periplasmic protein	**1.684**	(0.015)	**0.605**	(0.019)
	YPTB0339 *(hmuS)*	YPO0282	hemin transport protein	**1.577**	(0.021)	**0.666**	(0.038)
	YPTB0340 *(hmuR)*	YPO0283	hemin receptor precursoR	**7.426**	(< 0.001)		
	YPTB0343	YPO0285	conserved hypothetical protein	**1.774**	(0.009)		
	YPTB0354 *(terB)*	YPO0296	tellurite resistance protein			**0.504**	(0.002)
	YPTB0371	YPO0315	putative regulatory protein	**0.409**	(< 0.001)		
	YPTB0516 *(phnG)*	YPO3457	PhnG protein	**1.713**	(0.016)		
	YPTB0521	YPO3452	putative ABC transporter transporter. ATP-binding protein	**1.679**	(0.029)		
	YPTB0594	YPO0451	putative cation-transporting P-type ATPase	**0.653**	(0.046)		
	YPTB0662 *(thiP)*	YPO0521	thiamine transport system permease protein	**1.973**	(0.003)	**1.574**	(0.035)
	YPTB0739 *(fhuC)*	YPO3392	ferrichrome transport ATP-binding protein FhuC	**1.932**	(0.046)		
	YPTB0740 *(fhuD)*	YPO3391	ferrichrome-binding periplasmic protein precursoR	**1.835**	(0.021)		
	YPTB0790 *(yhjA)*	YPO3342	putative cytochrome C peroxidase	**0.479**	(< 0.001)		
	YPTB0811 *(katY)*	YPO3319	catalase-peroxidase	**0.602**	(0.008)	**2.344**	(< 0.001)
	YPTB0986	YPO3130	conserved hypothetical protein			**1.749**	(0.003)
	YPTB1246 *(katA)*	YPO1207	catalase	**0.324**	(< 0.001)		
	YPTB1341	YPO1310	putative periplasmic substrate-binding transport protein	**3.085**	(< 0.001)		
	YPTB1343 *(yiuC)*	YPO1312	putative siderophore ABC transporter. ATP-binding subunit	**2.467**	(< 0.001)		
	YPTB1409 *(focA)*	YPO1384	putative formate transporter 1	**0.563**	(0.005)	**1.638**	(0.013)
	YPTB1549 *(ysuR)*	YPO1537	putative iron-siderophore receptoR	**2.609**	(< 0.001)	**0.669**	(0.039)
	YPTB1659 *(ftnA)*	YPO1783	ferritin	**0.18**	(< 0.001)		
	YPTB1725	YPO1854	putative membrane protein	**2.084**	(0.013)		
	YPTB1939	YPO1941	putative membrane protein			**0.549**	(< 0.001)
	YPTB1940	YPO1942	putative exported protein	**1.776**	(0.003)		
	YPTB1947 *(tehB)*	YPO1949	putative tellurite resistance protein	**1.722**	(0.025)		
	YPTB2044 *(znuA)*	YPO2061	exported high-affinity zinc uptake system protein			**1.693**	(0.009)
	YPTB2052	YPO2069	putative integral membrane protein	**1.506**	(0.041)		
	YPTB2108 *(oppD)*	YPO2185	oligopeptide transport ATP-binding protein		(< 0.001)		
	YPTB2299 *(sodB)*	YPO2386	superoxide dismutase [Fe]		(< 0.001)		
	YPTB2347 *(yfeA)*	YPO2439	periplasmic-binding protein	**11.88**	(< 0.001)		
	YPTB2348 *(yfeB)*	YPO2440	ATP-binding transport protein	**3.141**	(< 0.001)		
	YPTB2349 *(yfeC)*	YPO2441	chelated iron transport system membrane protein	**4.375**	(< 0.001)		
	YPTB2350 *(yfeD)*	YPO2442	chelated iron transport system membrane protein	**2.104**	(< 0.001)		
	YPTB2546 *(dps)*	YPO2509	putative DNA-binding protein			**0.566**	(< 0.001)
	YPTB2682 *(yfuA)*	YPO2958	iron(III)-binding periplasmic protein	**2.387**	(< 0.001)		
	YPTB2743	YPO3025	conserved hypothetical protein			**1.598**	(0.049)
	YPTB2769 *(ydeN)*	YPO3047	putative sulfatase	**0.382**	(< 0.001)		
	YPTB2771	YPO3049	putative binding protein-dependent transport system. inner-membrane comp	**0.592**	(0.026)		
	YPTB2803 *(ppx)*	YPO2837	putative exopolyphosphatase			**0.564**	(< 0.001)
	YPTB2934	YPO2675	putative potassium channel protein	**0.236**	(< 0.001)		
	YPTB2936	YPO2673	putative nickel transport protein	**0.398**	(< 0.001)		
	YPTB2974	YPO1072	ABC transporter permease protein	**1.328**	(0.027)		
	YPTB2979 *(cutF)*	YPO1067	putative copper homeostasis lipoprotein	**1.794**	(0.001)		
	YPTB3068	YPO0819	putative carbonic anhydrase	**0.344**	(< 0.001)		
	YPTB3074	YPO0829	putative sulfatase			**1.622**	(0.023)
	YPTB3227	YPO0955	putative periplasmic substrate-binding transport protein	**3.085**	(< 0.001)		
	YPTB3298	YPO1011	putative TonB-dependent outer membrane receptoR	**2.15**	(0.004)	**0.554**	(0.022)
	YPTB3605 *(ssuA)*	YPO3624	aliphatic sulfonates binding protein (pseudogene. insertion)	**1.534**	(0.048)		
	YPTB3700 *(bfr)*	YPO0206	bacterioferritin			**0.66**	(0.003)
	YPTB3701 *(bfd)*	or2776	putative bacterioferritin-associated ferredoxin	**1.856**	(0.002)		
	YPTB3706	YPO0199	conserved hypothetical protein			**1.467**	(0.009)
	YPTB3737	YPO0164	putative membrane receptor protein (pseudogene. inframe insertion)			**0.568**	(0.001)
	YPTB3767 *(feoA)*	YPO0133	hypothetical ferrous iron transport protein A	**1.509**	(0.045)	**0.624**	(0.025)
	YPTB3857	YPO4022	putative iron transport protein	**3.127**	(< 0.001)		
	YPTB3858	YPO4023	putative iron transport permease	**2.236**	(< 0.001)		
	YPTB3860	YPO4025	putative iron ABC transporter. ATP-binding protein	**2.216**	(< 0.001)		
	YPTB3925 *(sodA)*	YPO4061	superoxide dismutase [Mn]	**3.101**	(< 0.001)	**0.613**	(0.017)
	YPTB3963 *(pstS)*	YPO4117	putative phosphate-binding periplasmic protein	**1.741**	(< 0.001)		
**Q: secondary metabolite biosynthesis, transport and catabolism**							
	YPTB1030 *(ybbP)*	YPO3078	putative permease	**1.661**	(0.022)	**1.756**	(0.012)
	YPTB1480	YPO1462	putative acyl carrier protein			**1.669**	(0.048)
	YPTB1544 *(ysuG)*	YPO1532	putative siderophore biosynthetic enzyme	**2.403**	(0.005)		
	YPTB1550	YPO1538	putative siderophore biosynthetic enzyme	**8.255**	(< 0.001)	**0.649**	(0.048)
	YPTB1596 *(irp2)*	YPO1911	yersiniabactin biosynthetic protein	**1.526**	(0.029)		
	YPTB1966 *(hutI)*	YPO1972	imidazolonepropionase	**0.632**	(0.004)		
	YPTB2064	YPO2082	putative fumarylacetoacetate hydrolase family protein			**0.73**	(0.018)
	YPTB2470 *(acpP)*	YPO1600	acyl carrier protein			**0.661**	(0.002)
	YPTB2471 *(fabG)*	YPO1599	3-oxoacyl-[acyl-carrier protein] reductase			**0.573**	(0.002)
	YPTB2561 *(menF)*	YPO2528	menaquinone-specific isochorismate synthase			**0.522**	(0.011)
	YPTB2626 *(fabB)*	YPO2757	3-oxoacyl-[acyl-carrier-protein] synthase I			**0.605**	(0.009)
	YPTB3258 *(yspI)*	YPO0984	N-acylhomoserine lactone synthase YspI			**0.494**	(0.032)
	YPTB3263 *(iucA)*	YPO0989	aerobactin synthetase (subunit alpha)	**3.789**	(< 0.001)		
	YPTB3265 *(iucC)*	YPO0992	aerobactin synthetase (subunit beta)	**2.601**	(< 0.001)		
	YPTB3266 *(iucD)*	YPO0993	putative siderophore biosynthesis protein IucD	**2.151**	(0.002)		
	YPTB3297	YPO0777	putative peptide/polyketide synthase subunit	**2.142**	(< 0.001)		
**R: general function prediction only**							
	YPTB0026	YPO0027	conserved hypothetical protein			**0.616**	(0.018)
	YPTB0057 *(tdh)*	YPO0060	threonine 3-dehydrogenase	**0.663**	(0.041)		
	YPTB0063 *(secB)*	YPO0067	protein-export protein			**0.683**	(0.012)
	YPTB0071 *(cpxP)*	YPO0075	putative exported protein			**0.337**	(< 0.001)
	YPTB0156	YPO3881	putative chaperone protein	**0.657**	(0.01)		
	YPTB0158	YPO3879	putative outer membrane usher protein			**1.626**	(0.016)
	YPTB0221 *(ftsY)*	YPO3814	cell division protein (pseudogene. inframe deletion)			**0.71**	(0.035)
	YPTB0257 *(aarF)*	YPO3779	ubiquinone biosynthesis protein	**0.68**	(0.048)		
	YPTB0258 *(tatA)*	YPO3778	Sec-independent protein translocase protein tatA	**0.685**	(0.003)		
	YPTB0327	YPO0270	putative type III secretion apparatus protein			**0.658**	(0.014)
	YPTB0331	YPO0274	putative integral membrane protein			**2.083**	(< 0.001)
	YPTB0353 *(terA)*	YPO0295	putative tellurite resistance protein			**0.565**	(< 0.001)
	YPTB0359	YPO0302	putative outer membrane fimbrial usher protein	**1.398**	(0.049)		
	YPTB0374 *(qor)*	YPO0319	quinone oxidoreductase	**1.393**	(0.04)		
	YPTB0448	YPO3528	putative exported protein	**0.451**	(< 0.001)		
	YPTB0466	YPO3510	putative membrane protein	**1.768**	(0.019)		
	YPTB0493 *(ibeB)*	YPO3481	probable outer membrane efflux lipoprotein	**1.863**	(0.015)		
	YPTB0576 *(osmY)*	YPO0431	osmotically inducible protein Y	**3.79**	(< 0.001)	**0.421**	(0.001)
	YPTB0706 *(hofB)*	YPO3426	putative type II secretion system protein	**0.711**	(0.043)		
	YPTB0808	YPO3322	conserved hypothetical protein	**0.555**	(0.031)		
	YPTB0832 *(corE)*	YPO3297	putative membrane protein	**1.401**	(0.011)		
	YPTB0839 *(dcuB)*	YPO3288	anaerobic C4-dicarboxylate transporter (pseudogene. F/S)	**0.442**	(0.001)		
	YPTB0878	or0629	5-methylthioribose kinase	**1.955**	(0.029)		
	YPTB0929 *(yajC)*	YPO3190	putative membrane protein	**1.526**	(0.047)		
	YPTB0965	YPO3151	conserved hypothetical protein			**1.553**	(0.047)
	YPTB1061 *(yapC)*	YPO2796	putaive autotransporter protein	**1.82**	(0.003)		
	YPTB1111	YPO2618	conserved hypothetical protein	**0.577**	(0.001)		
	YPTB1155	YPO1120	conserved hypothetical protein	**1.404**	(0.003)		
	YPTB1159 *(tolB)*	YPO1124	TolB colicin import protein	**1.46**	(0.025)		
	YPTB1194	YPO1163	putative membrane protein			**0.678**	(< 0.001)
	YPTB1210	or4494	possible ABC transporter multidrug efflux pump. permease subunit	**0.669**	(0.03)		
	YPTB1321	YPO1289	conserved hypothetical protein	**1.662**	(0.026)		
	YPTB1335 *(psaB)*	YPO1304	chaperone protein PsaB precursoR	**0.216**	(< 0.001)		
	YPTB1512	YPO1496	putative heme-binding protein	**2.456**	(< 0.001)		
	YPTB1513	YPO1497	ABC transporter ATP-binding protein	**1.878**	(0.009)	**0.453**	(0.001)
	YPTB1540 *(ysuF)*	YPO1528	putative ferric iron reductase	**4.019**	(< 0.001)		
	YPTB1646 *(hpaC)*	YPO1770	4-hydroxyphenylacetate 3-monooxygenase coupling protein	**1.613**	(0.023)		
	YPTB1660	YPO1784	putative copper resistance protein	**3.934**	(< 0.001)	**0.703**	(0.017)
	YPTB1680 *(flgJ)*	YPO1807	flagellar protein FlgJ			**1.906**	(0.01)
	YPTB1693	YPO1820A				**2.053**	(0.011)
	YPTB1695 *(fliN)*	YPO1822	flagellar motor switch protein FliN			**1.79**	(0.021)
	YPTB1728 *(wrbA)*	YPO1857	trp repressor binding protein	**1.401**	(0.006)		
	YPTB1733 *(ydgC)*	YPO1863	putative membrane protein	**0.626**	(0.001)		
	YPTB1919	YPO1920	probable fimbrial usher protein			**1.461**	(0.019)
	YPTB1944	YPO1946	ABC transporter. ATP-binding protein	**1.957**	(0.003)	**1.588**	(0.03)
	YPTB1985	YPO1993	putative dehydrogenase			**1.586**	(0.007)
	YPTB2019	YPO2037	conserved hypothetical protein	**1.56**	(0.021)		
	YPTB2101 *(hns)*	YPO2175	Hns DNA binding protein			**0.405**	(0.001)
	YPTB2169	or1554	putative toxin transport protein (pseudogene. F/S)	**1.549**	(0.027)		
	YPTB2289	YPO2375	putative aldo/keto reductase	**1.508**	(0.006)		
	YPTB2291	YPO2377	putative membrane protein	**0.607**	(0.018)	**1.529**	(0.038)
	YPTB2345 *(marC)*	YPO2437	multiple antibiotic resistance protein	**1.771**	(0.022)		
	YPTB2368 *(ogl)*	YPO1713	oligogalacturonate lyase	**0.699**	(0.045)		
	YPTB2390	YPO1689	putative lipoprotein	**1.312**	(0.032)		
	YPTB2452 *(ycfL)*	YPO1612	putative lipoprotein	**1.45**	(0.046)		
	YPTB2459	or3719	hypothetical	**0.379**	(< 0.001)		
	YPTB2471 *(fabG)*	YPO1599	3-oxoacyl-[acyl-carrier protein] reductase			**0.573**	(0.002)
	YPTB2488	YPO2451	conserved hypothetical protein			**0.671**	(0.026)
	YPTB2492	or3693	conserved hypothetical protein	**0.602**	(0.009)		
	YPTB2553	or3647	conserved hypothetical protein	**0.573**	(0.006)		
	YPTB2604	or3598	conserved hypothetical (pseudogene. F/S)	**1.86**	(0.004)		
	YPTB2646 *(ccmD)*	or3557	putative heme exporter protein D	**0.36**	(< 0.001)		
	YPTB2722	YPO3001	putative pyridine nucleotide-disulphide oxidoreductase			**1.897**	(0.04)
	YPTB2723	YPO3002	putative permease	**1.328**	(0.036)		
	YPTB2727	YPO3007	putative membrane protein	**1.932**	(0.008)		
	YPTB2753	YPO3031	putative acetyltransferase	**1.391**	(0.02)		
	YPTB2837 *(engA)*	YPO2875	putative GTP-binding protein	**0.573**	(0.015)		
	YPTB2843	YPO2881	putative fimbrial biogenesis protein			**0.686**	(0.022)
	YPTB2891 *(lepB)*	YPO2717	signal peptidase I	**1.527**	(0.032)	**1.518**	(0.035)
	YPTB2902	YPO2706	conserved hypothetical protein	**0.549**	(0.026)	**1.898**	(0.018)
	YPTB3116	or3203	hypothetical protein			**0.556**	(0.022)
	YPTB3176	YPO0900	putative hemolysin III			**1.415**	(0.038)
	YPTB3223	YPO0951	Putative methyltransferase			**0.612**	(0.023)
	YPTB3238	YPO0966	putative kinase	**0.505**	(0.032)		
	YPTB3285	or3082	Putative autotransporter secreted protein			**1.593**	(0.028)
	YPTB3291	YPO0771	ABC-transporter transmembrane protein	**1.705**	(0.014)		
	YPTB3357	YPO0704	flagellar assembly protein	**0.611**	(0.049)		
	YPTB3381	YPO0684	putative membrane protein			**1.67**	(0.014)
	YPTB3382 *(exbD)*	YPO0683	ExbD/TolR-family transport protein	**9.812**	(< 0.001)		
	YPTB3383 *(exbB)*	YPO0682	MotA/TolQ/ExbB proton channel family protein	**4.164**	(< 0.001)		
	YPTB3388	YPO0676	putative aldo/keto reductase family protein			**1.632**	(0.042)
	YPTB3438	YPO0617	putative membrane protein	**1.993**	(0.004)		
	YPTB3464	YPO0595	conserved hypothetical protein	**0.528**	(0.005)		
	YPTB3493	YPO3548	putative exported protein	**0.602**	(0.005)		
	YPTB3496	YPO3551	putative exported protein			**0.553**	(< 0.001)
	YPTB3558 *(tldD)*	YPO3672	putative modulator of DNA gyrase			**0.702**	(0.025)
	YPTB3568	YPO3662	conserved hypothetical protein			**1.489**	(0.008)
	YPTB3659	YPO0247	putative transferase	**1.289**	(0.029)		
	YPTB3745 *(gph)*	YPO0156	phosphoglycolate phosphatase	**0.596**	(0.022)		
	YPTB3757	YPO0144	putative hydrolase	**1.58**	(0.013)		
	YPTB3879	or2640	possible type I restriction enzyme (restriction subunit)	**1.554**	(0.044)		
	YPTB3896	YPO4044	fimbrial protein			**1.644**	(0.021)
	YPTB3939	YPO4093	putative haloacid dehalogenase-like hydrolase	**1.753**	(0.02)		
	YPTB3948 *(yidC)*	YPO4102	probable membrane protein	**0.666**	(0.024)	**0.645**	(0.016)
	YPTB3953 *(yieG)*	YPO4107	Xanthine/uracil permeases family protein	**0.638**	(0.046)	**1.583**	(0.042)
**S: function unknown**							
	YPTB0015 *(mobA)*	YPO0013A	molybdopterin-guanine dinucleotide biosynthesis protein A			**0.703**	(0.032)
	YPTB0020	YPO0020	conserved hypothetical protein	**0.493**	(< 0.001)		
	YPTB0040	YPO0043	conserved hypothetical protein	**0.705**	(0.029)	**0.656**	(0.011)
	YPTB0089	YPO0093	conserved hypothetical protein	**1.682**	(< 0.001)	**0.681**	(0.008)
	YPTB0196	or0133	conserved hypothetical protein			**3.061**	(< 0.001)
	YPTB0219	YPO3816A				**1.383**	(0.046)
	YPTB0296	YPO3732	conserved hypothetical protein			**0.558**	(< 0.001)
	YPTB0378	YPO0323	conserved hypothetical protein	**1.483**	(0.016)		
	YPTB0454	YPO3522	conserved hypothetical protein	**1.472**	(0.03)		
	YPTB0478	YPO3498	conserved hypothetical protein	**0.734**	(0.038)		
	YPTB0506	or0367	conserved hypothetical protein	**0.541**	(0.013)		
	YPTB0547	YPO0407	conserved hypothetical protein			**1.999**	(< 0.001)
	YPTB0589	YPO0445	conserved hypothetical protein			**1.579**	(0.007)
	YPTB0600 *(creA)*	YPO0457	putative exported protein	**1.924**	(< 0.001)		
	YPTB0627	YPO0485	putative membrane protein			**1.523**	(0.018)
	YPTB0639	YPO0498	hypothetical protein			**0.302**	(< 0.001)
	YPTB0640	YPO0499	hypothetical protein				(< 0.001)
	YPTB0641	YPO0500	conserved hypothetical protein				(< 0.001)
	YPTB0642	YPO0501	conserved hypothetical protein			**0.346**	(< 0.001)
	YPTB0643	YPO0502	conserved hypothetical protein				(< 0.001)
	YPTB0644	YPO0503	conserved hypothetical protein			**0.702**	(0.012)
	YPTB0646	YPO0505	conserved hypothetical protein			**0.355**	(< 0.001)
	YPTB0648	YPO0507	conserved hypothetical protein			**0.679**	(0.044)
	YPTB0649a	YPO0508	hypothetical protein			**0.56**	(0.031)
	YPTB0650	YPO0510	hypothetical protein	**1.447**	(0.043)	**0.368**	(< 0.001)
	YPTB0653	YPO0512	putative lipoprotein	**1.45**	(0.035)	**0.618**	(0.009)
	YPTB0654	YPO0513	conserved hypothetical protein			**0.364**	(0.002)
	YPTB0655	YPO0514	putative OmpA-family membrane protein			**0.383**	(< 0.001)
	YPTB0679	YPO0546	conserved hypothetical protein	**1.35**	(0.036)		
	YPTB0701	YPO3431	conserved hypothetical protein	**0.663**	(< 0.001)		
	YPTB0744	YPO3387	conserved hypothetical protein	**2.792**	(< 0.001)		
	YPTB0876	or0627	methionine salvage pathway enzyme E-2/E-2'			**1.533**	(0.007)
	YPTB0976 *(ybaY)*	YPO3140	putative lipoprotein			**1.504**	(0.043)
	YPTB1021	YPO3087	conserved hypothetical protein			**1.394**	(0.029)
	YPTB1057	YPO2801	putative membrane protein	**1.682**	(0.044)		
	YPTB1078	YPO2585	putative carbohydrate kinase			**1.704**	(0.035)
	YPTB1085	YPO2592	putative membrane protein			**0.609**	(0.015)
	YPTB1161	YPO1126	putative exported protein			**0.596**	(0.004)
	YPTB1215	YPO1174	hypothetical protein			**1.527**	(0.047)
	YPTB1222	YPO1181	putative membrane protein			**0.697**	(0.032)
	YPTB1227	YPO1186	conserved hypothetical protein	**0.634**	(0.005)		
	YPTB1297	YPO1261	conserved hypothetical protein			**0.659**	(0.014)
	YPTB1387	YPO1361	putative membrane protein	**1.593**	(0.003)		
	YPTB1389	YPO1363	putative virulence factoR	**0.632**	(0.043)		
	YPTB1422	YPO1397	conserved hypothetical protein (pseudogene. inframe deletion)			**1.803**	(0.012)
	YPTB1432	YPO1408	putative exported protein	**0.671**	(0.044)		
	YPTB1499	YPO1483	hypothetical protein	**1.491**	(0.029)		
	YPTB1504	YPO1487	conserved hypothetical protein	**2.062**	(0.005)		
	YPTB1571	YPO1560	conserved hypothetical protein	**1.34**	(0.036)		
	YPTB1640 *(hpaD)*	YPO1764	3.4-dihydroxyphenylacetate 2.3-dioxygenase	**0.625**	(0.046)		
	YPTB1729	YPO1858	putative exported protein	**0.704**	(0.037)		
	YPTB1901	or1366	conserved hypothetical protein	**1.511**	(0.01)		
	YPTB1902	YPO1882	conserved hypothetical protein	**1.552**	(< 0.001)		
	YPTB1941	YPO1943	putative membrane protein	**2.118**	(0.004)		
	YPTB2085	YPO2159	conserved hypothetical protein	**0.606**	(0.032)		
	YPTB2146	YPO2224	putative membrane protein			**1.568**	(0.018)
	YPTB2214	YPO2291	putative virulence factoR	**0.638**	(0.023)		
	YPTB2234	YPO2315	putative exported protein	**1.503**	(0.008)		
	YPTB2265	YPO2347	putative membrane protein	**1.284**	(0.04)		
	YPTB2314	YPO2404	conserved hypothetical protein	**2.602**	(< 0.001)		
	YPTB2352	YPO2444	conserved hypothetical protein	**0.704**	(0.017)		
	YPTB2388	YPO1693	conserved hypothetical protein	**1.569**	(0.038)		
	YPTB2444 *(ycfJ)*	YPO1624	putative exported protein	**2.296**	(0.001)		
	YPTB2481	YPO1588	conserved hypothetical protein			**0.759**	(0.035)
	YPTB2526	YPO2489	conserved hypothetical protein			**1.361**	(0.02)
	YPTB2547	YPO2510	putative exported protein	**0.31**	(< 0.001)		
	YPTB2594	YPO2563	conserved hypothetical protein	**0.563**	(< 0.001)		
	YPTB2638	YPO2745	conserved hypothetical protein	**0.483**	(0.001)		
	YPTB2651 *(lemA)*	YPO2732	putative exported protein	**0.381**	(< 0.001)		
	YPTB2660	YPO2724	putative membrane protein	**1.486**	(0.038)		
	YPTB2661	YPO2723	possible OmpA family (pseudogene. IS100 insertion)			**1.796**	(0.048)
	YPTB2674	YPO2949	hypothetical protein	**1.753**	(0.045)		
	YPTB2693	YPO2970	putative lipoprotein			**0.65**	(0.049)
	YPTB2694	YPO2971	putative lipoprotein			**0.454**	(< 0.001)
	YPTB2745 *(ygiW)*	YPO3027	putative exported protein			**0.648**	(0.002)
	YPTB2907	YPO2701	putative membrane protein			**0.658**	(0.038)
	YPTB2981	YPO1065	conserved hypothetical protein			**0.721**	(0.035)
	YPTB3117	YPO0874	hypothetical protein	**0.675**	(0.039)		
	YPTB3161	or2087	Hypothetical bacteriophage protein.			**0.64**	(0.033)
	YPTB3186	YPO0911	putative exported protein			**1.578**	(0.023)
	YPTB3187	YPO0912	conserved hypothetical protein			**0.612**	(0.006)
	YPTB3206	YPO0934	conserved hypothetical protein	**0.69**	(0.039)		
	YPTB3222	YPO0950	conserved hypothetical protein			**0.547**	(0.006)
	YPTB3301	or2158	putative antigenic leucine-rich repeat protein			**0.581**	(0.004)
	YPTB3403	YPO0659	conserved hypothetical protein	**0.768**	(0.028)	**0.753**	(0.019)
	YPTB3429	YPO0626	Conserved hypothetical	**0.619**	(0.034)		
	YPTB3468 *(hdeD)*	YPO0590	putative membrane protein	**0.209**	(< 0.001)		
	YPTB3484	YPO0572	putative exported protein			**0.533**	(0.013)
	YPTB3485 *(yqjD)*	YPO0570	putative membrane protein	**0.674**	(0.02)	**0.576**	(0.002)
	YPTB3486	YPO0569A		**0.606**	(0.026)		
	YPTB3510	YPO3565	putative membrane protein			**0.745**	(0.02)
	YPTB3573 *(panF)*	YPO3657A	sodium/pantothenate symporteR			**0.732**	(0.028)
	YPTB3581	YPO3649	putative gamma carboxymuconolactone decarboxylase	**0.573**	(0.01)		
	YPTB3617	or2852	putative Rhs accessory genetic element	**0.632**	(0.037)		
	YPTB3622	YPO3607	conserved hypothetical protein			**0.589**	(0.014)
	YPTB3748	YPO0153	conserved hypothetical membrane protein			**0.614**	(0.033)
	YPTB3773	YPO0127	conserved hypothetical protein	**2.928**	(< 0.001)		
	YPTB3897	YPO4045	putative membrane protein			**0.609**	(0.015)
**T: signal transduction mechanisms**							
	YPTB0022 *(ntrC)*	YPO0022	nitrogen regulation protein			**0.723**	(0.037)
	YPTB0035 *(spoT)*	YPO0038	guanosine-3'.5'-bisbis(diphosphate) 3'-pyrophosphydrolase	**0.684**	(0.011)		
	YPTB0071 *(cpxP)*	YPO0075	putative exported protein			**0.337**	(< 0.001)
	YPTB0356 *(terD)*	YPO0298	tellurium resistance protein	**1.485**	(0.034)		
	YPTB0357 *(terE)*	YPO0299	tellurium resistance protein	**1.501**	(0.005)	**0.72**	(0.019)
	YPTB0468 *(basS)*	YPO3508	two-component system sensor protein			**2.184**	(0.001)
	YPTB0541	YPO0401	putative transcriptional regulatoR	**0.652**	(0.035)		
	YPTB0570 *(hmsT)*	YPO0425	HmsT protein	**0.574**	(0.014)	**1.64**	(0.026)
	YPTB0592	YPO0449	putative exported protein			**0.591**	(0.022)
	YPTB0601 *(arcA)*	YPO0458	aerobic respiration control protein	**0.465**	(0.001)		
	YPTB0734 *(dksA)*	YPO3397	DnaK suppressor protein homologue			**0.508**	(< 0.001)
	YPTB0789	YPO3343	probable extracellular solute-binding protein	**0.462**	(0.001)		
	YPTB1108 *(glnH)*	YPO2615	putative amino acid-binding protein precursoR	**0.564**	(0.005)		
	YPTB1258 *(rcsB)*	YPO1218	probable two component response regulator component B			**0.693**	(0.016)
	YPTB1259	YPO1219	putative two component sensor kinase			**0.696**	(0.041)
	YPTB1922	YPO1923	Putative sensor protein			**1.451**	(0.044)
	YPTB1957 *(narX)*	YPO1959	nitrate/nitrite sensor protein	**1.83**	(0.031)		
	YPTB2099	YPO2173	probable response regulatoR			**1.533**	(0.043)
	YPTB2156 *(cstA)*	YPO2234	putative carbon starvation protein A	**0.544**	(0.012)	**0.624**	(0.045)
	YPTB2222 *(fnr)*	YPO2300	fumarate and nitrate reduction regulatory protein	**0.699**	(0.001)	**1.584**	(< 0.001)
	YPTB2230 *(rstA)*	YPO2308	two-component regulatory system. response regulator protein	**0.658**	(0.024)		
	YPTB2378	YPO1703	conserved hypothetical protein			**0.711**	(0.022)
	YPTB2396 *(cheZ)*	YPO1681	chemotaxis protein CheZ			**1.477**	(0.027)
	YPTB2405 *(cheA)*	YPO1666	chemotaxis protein CheA	**1.725**	(0.015)		
	YPTB2435 *(phoQ)*	YPO1633	sensor protein kinase	**0.527**	(< 0.001)		
	YPTB2548 *(glnH)*	YPO2511	putative glutamine-binding periplasmic protein			**1.797**	(0.014)
	YPTB2635 *(sixA)*	YPO2748	putative phosphohistidine phosphatase			**1.508**	(0.002)
	YPTB2763 *(narP)*	YPO3041	nitrate/nitrite response regulator protein NarP			**0.715**	(0.043)
	YPTB2894 *(rseC)*	YPO2714	sigma E factor regulatory protein			**1.431**	(0.034)
	YPTB2895 *(rseB)*	YPO2713	sigma E factor regulatory protein			**1.466**	(0.04)
	YPTB2896 *(rseA)*	YPO2712	sigma E factor negative regulatory protein			**1.652**	(< 0.001)
	YPTB3350 *(fleR)*	YPO0712	sigma-54 transcriptional regulatory protein	**2.134**	(0.004)		
	YPTB3408 *(glnE)*	YPO0653	glutamate-ammonia-ligase adenylyltransferase	**1.792**	(0.046)		
	YPTB3410	YPO0651	putative exported protein	**1.988**	(< 0.001)		
	YPTB3463 *(terX)*	YPO0596	putative tellurium resistance protein	**0.522**	(< 0.001)		
	YPTB3500 *(arcB)*	YPO3555	aerobic respiration control sensor/response regulatory protein	**0.674**	(0.012)		
	YPTB3566 *(yhdA)*	YPO3664	putative exported protein			**1.836**	(< 0.001)
	YPTB3729 *(crp)*	YPO0175	cAMP-regulatory protein			**0.641**	(< 0.001)
	YPTB3812 *(uspA)*	YPO3970	universal stress protein A	**0.291**	(< 0.001)	**1.554**	(0.038)
	YPTB3847 *(uhpA)*	YPO4012	two-component system response regulatoR			**2.004**	(0.006)
	YPTB3957	YPO4111	putative periplasmic solute-binding protein	**1.59**	(0.006)		
	YPTB2341 *(infC)*	YPO2432	translation initiation factor IF-3			**0.539**	(< 0.001)

**Table 3:** *Y. pseudotuberculosis *IP32953 pYV plasmid-harbored genes that are transcriptionally regulated by growth medium and/or temperature.

			**Fold ratio in gene transcription **(*p*-value)			
			
**Gene designation**	**Genoscript spot ID**	**Gene product/function**	**Human plasma/Luria Bertani Broth**		**37°C/28°C**	
pYV0013	pCD1-yadA	hypothetical protein	**13.528**	(< 0.001)	**1.577**	(0.046)
pYV0014	pCD1-AAC62595	possible transposase remnant	**1.789**	(0.025)	**1.884**	(0.016)
pYV0017	pCD1-tnpR	putative resolvase	**0.341**	(< 0.001)		
pYV0020	pCD1-sycH	putative YopH targeting protein	**5.088**	(< 0.001)	**2.715**	(< 0.001)
pYV0024	pCD1-sycE	putative YopE chaperone	**5.781**	(< 0.001)	**2.403**	(0.005)
pYV0040	pCD1-yopK/yopQ	Yop targeting protein YopK, YopQ			**1.666**	(0.019)
pYV0047	pCD1-yopM	putative targeted effector protein	**3.468**	(< 0.001)	**2.252**	(0.002)
pYV0054	pCD1-yopD	putative Yop negative regulation/targeting component	**2.933**	(< 0.001)	**2.211**	(0.004)
pYV0055	pCD1-yopB	putative Yop targeting protein	**2.816**	(< 0.001)	**2.196**	(< 0.001)
pYV0056	pCD1-lcrH	low calcium response protein H	**3.522**	(< 0.001)	**1.851**	(0.021)
pYV0057	pCD1-lcrV	putative V antigen, antihost protein/regulator	**1.904**	(< 0.001)	**1.637**	(0.003)
pYV0058	pCD1-lcrG	putative Yop regulator	**1.713**	(0.014)		
pYV0059	pCD1-lcrR	hypothetical protein LcrR	**1.469**	(0.001)	**1.849**	(< 0.001)
pYV0062	pCD1-yscX	putative type III secretion protein	**1.629**	(0.009)	**1.533**	(0.020)
pYV0065	pCD1-yopN	putative membrane-bound Yop targeting protein	**2.612**	(< 0.001)	**1.549**	(0.049)
pYV0067	pCD1-yscN	putative Yops secretion ATP synthase	**2.856**	(< 0.001)	**1.717**	(0.002)
pYV0068	pCD1-yscO	putative type III secretion protein	**3.088**	(< 0.001)		
pYV0069	pCD1-yscP	putative type III secretion protein			**1.593**	(0.018)
pYV0070	pCD1-yscQ	putative type III secretion protein	**1.635**	(0.026)		
pYV0071	pCD1-yscR	putative Yop secretion membrane protein	**1.949**	(0.012)		
pYV0072	pCD1-yscS	putative type III secretion protein	**2.331**	(< 0.001)	**1.897**	(0.002)
pYV0075	pCD1-virG	putative Yop targeting lipoprotein	**1.957**			
pYV0076	pCD1-lcrF/virF	putative thermoregulatory protein	**1.441**	(0.042)		
pYV0078	pCD1-yscB	hypothetical protein	**3.038**	(0.002)		
pYV0079	pCD1-yscC	putative type III secretion protein	**2.384**	(0.001)	**2.713**	(< 0.001)
pYV0080	pCD1-yscD	putative type III secretion protein	**2.542**	(< 0.001)	**1.990**	(0.005)
pYV0081	pCD1-yscE	putative type III secretion protein	**2.711**	(0.001)	**1.809**	(0.032)
pYV0082	pCD1-yscF	putative type III secretion protein	**2.117**	(0.017)		
pYV0083	pCD1-yscG	putative type III secretion protein	**2.463**	(< 0.001)		
pYV0085	pCD1-yscI	putative type III secretion protein	**1.864**	(0.003)		
pYV0089	pCD1-yscM	putative type III secretion regulatory protein	**0.679**	(0.036)		

**Figure 1:**
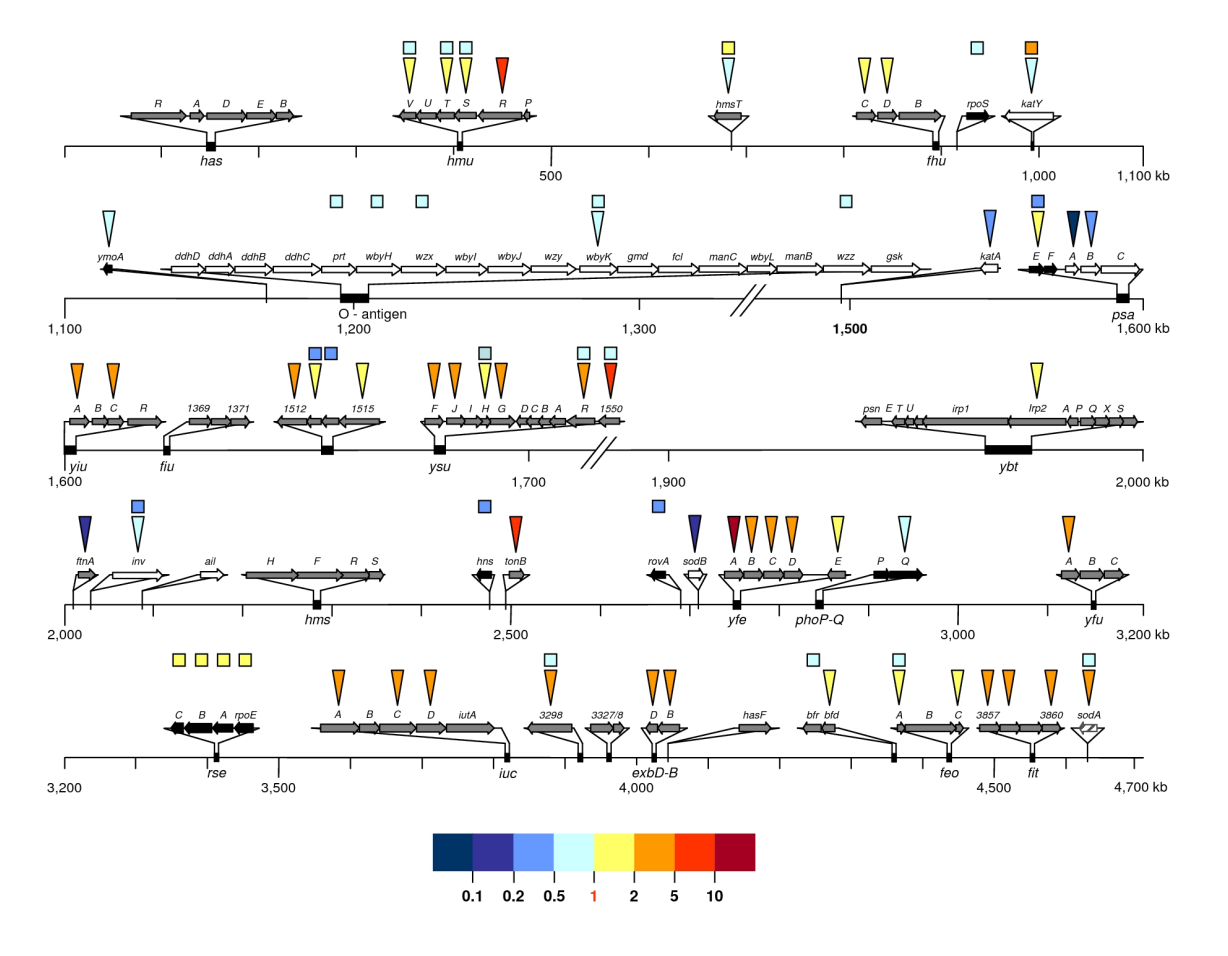
**Medium- and temperature-dependent differential expression of *Y. pseudotuberculosis *chromosomal genes involved in virulence and/or iron uptake & storage**. Significant (*p *< 0.05) upshifts (yellow to red scale) or downshifts (blue scale) in individual gene transcription levels when bacteria were grown in human plasma versus LB (triangles) and/or at 37°C versus 28°C (squares) are indicated by the color scale bar. Genes encoding iron uptake/storage systems, virulence factors and their regulators are symbolized by gray, white and black arrows, respectively. Nomenclature used for gene designation correspond to the *Y. pseudotuberculosis *IP32953 genome annotation. Mean fold changes in transcription and *p*-values are indicated in Table 2.

Free iron limitation is a well-known stimulus encountered by bacteria in plasma [10,11]. As expected, IP32953 genes required for iron storage (such as the ferritin-encoding gene *ftnA *[12] (Fig. 1)) were found to be downregulated in plasma. Transcriptional upregulation of most iron uptake systems (along with accessory protein-encoding genes *tonB*, *exbB *and *exbD*) (Fig. 1) is also consistent with this condition and is in agreement with the recent findings in *Y. pestis *[8]. As iron is used as a cofactor by numerous enzymes (mostly when complexed with sulfur), the metal is essential for a broad range of metabolic processes. Besides activation of iron homeostasis systems, lack of iron is also expected to be associated with a dramatic decrease in the transcription of genes encoding such enzymes, with the underlying goal of lowering iron consumption. This situation is exemplified by the *katA *gene that encodes catalase (a ferric enzyme involved in oxidative stress defense), whose transcription is decreased in both *Y. pestis *and *Y. pseudotuberculosis *during growth in plasma (Fig. 1). However, the increase in transcription of the *bio *locus (required for biotin synthesis [13]) and observed in both species) suggests that differential genetic control of a subset of iron-dependent enzymes may favor supply of this metal to the pathways that are most important for bacterial survival (and thus presumably at the expense of other, less critical ones). Furthermore, the impact of transcriptional downregulation on reorientation of metabolic fluxes may be minimized by the concomitant activation of genes coding for isoenzymes that are better suited to this situation.

One example is that of the manganese- and iron-dependent superoxide dismutase genes (i.e *sodA *and *sodB*), which are Fur-activated and -repressed, respectively (Fig. 1) in both *Y. pestis *and *Y. pseudotuberculosis*. Similarly, the class Ib ribonucleotide reductase (RNR)-encoding genes (*nrdHIEF*) are probably important for bacterial life in plasma, since they were found to be upregulated at the expense of those in classes III (*nrdDG*) and Ia (*nrdAB*) (Table 2) – even though all three classes are equally involved in generating the synthetic precursors for DNA. The fact that only the first class is Fur-activated [14] is consistent with this observation. Similar variations have also been recorded in *Y. pestis *[8]. However, whereas purine/pyrimidine metabolism has been shown to be essential for *Y. pestis *virulence [15], the role of this metabolic pathway in the physiopathology of *Y. pseudotuberculosis *has not yet been investigated. Along with class 1b RNRs, more than half of the enzymes in the tricarboxylic acid cycle (TCA) are known to be catalytically iron-dependent and/or believed to be transcriptionally activated by Fur [16]. Accordingly, and in line with transcriptome data from *Y. pestis*, we observed that transcription of these genes fell significantly when *Y. pseudotuberculosis *was grown in plasma.

**Figure 2:**
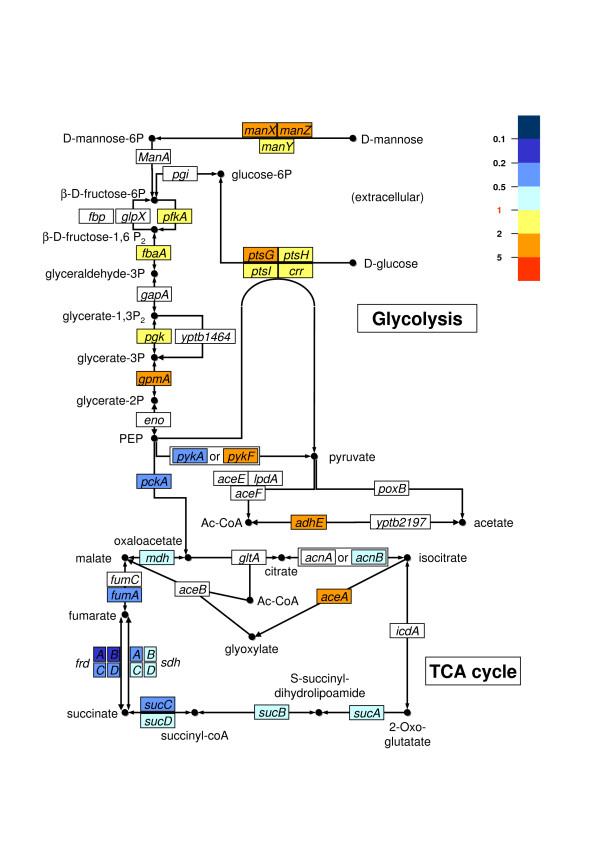
**Medium-dependent differential expression of genes coding for enzymes putatively involved in *Y. pseudotuberculosis *glycolysis and the tricarboxylic acid cycle (TCA cycle)**. Significant (*p *< 0.05) upshifts (yellow to red scale) or downshifts (blue scale) in individual gene transcription levels in human plasma versus LB is indicated by the color scale bar. Open boxes indicate genes whose expression levels did not vary significantly (*p *> 0.05). Although considered as not significant by statistical analysis of macroarray data (*p *= 0.053), transcriptional upregulation of *aceB *in human plasma was confirmed by qRT-PCR. Abbreviations: Ac-CoA: acetyl coenzyme A; PEP: phosphoenolpyruvate. Mean fold changes in transcription and *p*-values are indicated in Table 2.

In contrast to the low availability of iron in blood, glucose is readily available in this biological fluid and at a higher concentration (approx. 7 mM) than in LB broth. When *Y. pseudotuberculosis *was cultured in plasma, genes involved in glycolysis and the upstream, sugar-supplying, phosphoenolpyruvate-dependent systems were found to be upregulated, as depicted in Fig. 2. This finding is reminiscent of an aerobic phenomenon referred to as "glucose overflow metabolism"; this consists in channeling the carbon flow towards acetate formation instead of citrate formation, in order to prevent the excessive accumulation of NADH that would otherwise result from very high glucose consumption rates [17]. However, one main feature of glucose overflow in *E. coli *is acetate accumulation due to a strong transcriptional repression of the glyoxylate shunt *aceBAK *operon [18]. Interestingly, at least the first two of these genes are not down- but are up-regulated in *Y. pseudotuberculosis *(Fig. 2, Additional file 1), suggesting a need for this species to limit acetate overloads. The continuous de-repression of these genes (due to inactivation of the IclR repressor) suggests that this might also be the case in *Y. pestis*. These pathways are controlled by complex and finely balanced networks involving numerous pleiotropic regulators, including Fur, Crp, Fnr and ArcA [16,19]. This unexpected upregulation may well result from the combination of both high glucose and low iron levels in plasma. Whether this occurs through the strong transcriptional repression observed with both *fnr *and *arcA *remains to be addressed in future experiments.

Temperature upshift is typically considered to be the main signal indicating to bacteria that they have entered the host; this hypothesis is supported by the thermal dependency of almost all *Y. pseudotuberculosis *virulence genes and also many of the latter's regulators [3]. Several of these genes were also found to be influenced by growth in plasma and the changes were sometimes in the opposite direction to those seen with temperature upshifts: whereas expression of the invasin-encoding gene *inv *was significantly repressed during bacterial growth under both conditions, transcription of *psaA *(coding for the pH6 antigen) was promoted by temperature upshifts [6,20], but was one of the most strongly repressed in plasma. Interestingly, the impact of this medium on *psaA *transcription was not considered to be significant in *Y. pestis *and suggests that the pH6 antigen does not have the same importance in blood dissemination in the two species. In contrast to the latter two adhesins, transcriptional activation of *yadA *(harbored by the pYV plasmid and involved in adhesion) was found to be the highest of all the *Y. pseudotuberculosis *genes induced under plasma growth conditions. This observation is consistent with YadA's involvement in microbial resistance to complement [21,22]. Similarly, *ompC *whose product is believed to be targeted by lactoferricin [23], a bactericidal peptide derived from lactoferrin by enzymatic cleavage [24], is strongly repressed, whilst no significant modification was observed for the outer membrane-encoding genes *ompA *and *ompC*2.

**Figure 3:**
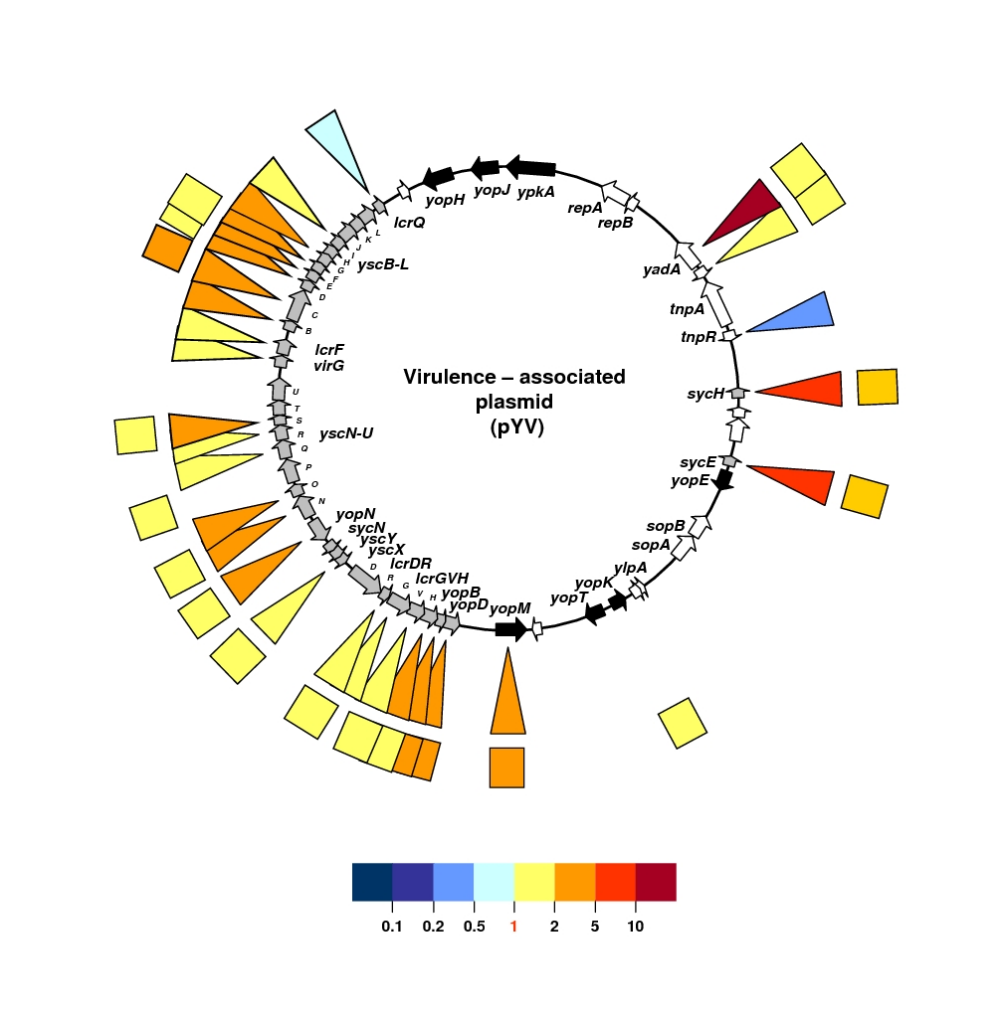
**Medium- and temperature-dependent differential expression of genes harbored by the *Y. pseudotuberculosis *virulence plasmid pYV**. Significant (*p *< 0.05) upshifts (yellow to red scale) or downshifts (blue scale) in individual gene transcription levels when bacteria were grown in human plasma versus LB (triangles) and/or at 37°C versus 28°C (squares) are indicated by the color scale bar. Only genes spotted on the macroarray (56 out of 99 pYV-borne genes) are shown and those encoding the secretion apparatus and Yop effectors are represented by grey and black boxes, respectively. Mean fold changes in transcription and *p*-values are indicated in Table 3.

Lastly, an essential determinant of bacterial virulence is the plasmid-encoded type III secretion system (TTSS) which performs intracellular delivery of a set of *Yersinia *outer proteins (Yops) that subvert the host's defenses [25]. Interestingly, *Y. pseudotuberculosis *growth in plasma induced the upregulation of 25 genes required for secretion, translocation and chaperoning of the Yop effector proteins in a similar fashion to that observed upon temperature upshift (Fig. 3). Furthermore, the apparently coordinated regulation of *yadA *and the TTSS-encoding genes by temperature and growth in plasma suggests the involvement of a common means of genetic control. YmoA (a chromatin-associated (histone-like) protein which is very similar in structure and function to the haemolysin expression modulating protein Hha from *Escherichia coli*) was shown to negatively influence YadA and Yop expression by favoring supercoiling of the pYV plasmid [26]. A two-fold reduction in *ymoA *transcription in plasma may be enough to contribute to the TTSS upregulation recorded in *Y. pseudotuberculosis*. Strikingly, this plasma-induced TTSS activation was not observed in *Y. pestis*, since only 3 out of the 25 genes mentioned above were found to be upregulated (in line with the statistically non-significant downregulation of *ymoA*); this raises the possibility that these two pathogenic *Yersinia *species may differ in their transcriptional regulation of pYV-harbored virulence genes.

## Conclusion

Overall transcription profiling of *Y. pseudotuberculosis *grown in an environment mimicking the blood stage of the infectious process revealed gene regulations that could not be anticipated from the results of previously reported single-stimulus studies. Our findings thus provide insight into how a number of simultaneously sensed environmental cues may be taken into account by the bacterium in a hierarchical manner. Furthermore, comparison of our analyses with those previously performed in *Y. pestis *suggests that transcription of common critical virulence factors may be differently influenced (at least in part) by the plasma environment in these two species.

## Methods

### DNA macroarray construction

Pairs of specific oligonucleotide primers were designed with the Primer 3 software for each of the 3,951 *Y. pseudotuberculosis *IP32953 CDSs. In order to avoid cross-hybridization, the specificity of the PCR products relative to the complete genome sequence was tested with CAAT-box software [27]. Primers purchased from Eurogentec were chosen in order to specifically amplify a ≈ 400 to 500 base pair (bp) fragment of each open reading frame (ORF), with a melting temperature of 51 to 60°C. Amplification reactions were performed in 96-well plates (Perkin-Elmer) in a 100 μl reaction volume containing 100 ng of *Y. pseudotuberculosis *IP32953 DNA, DNA polymerase (Dynazyme, New England Biolabs), 10 μM of each primer and 2 mM dNTPs (Perkin-Elmer). Reactions were cycled 45 times (94°C for 30 s; 60°C for 30 s; 72°C for 60 s) with a final cycle of 72°C for 7 min in a thermocycler. Each PCR product was checked by agarose gel electrophoresis and when DNA amplification was unsuccessful, PCR was repeated with another primer set. Overall, 3,951 of the 3,994 CDSs (98%) identified in the *Y. pseudotuberculosis *IP32953 genome were successfully amplified under our experimental conditions. ORF-specific PCR products, luciferase DNA (10 to 100 ng) and total genomic DNA from strain IP32953 were spotted onto 22 × 7-cm nylon membranes (Genetix) using a Qpix robot (Genetix). Immediately following spot deposition, membranes were immersed for 15 min in 0.5 M NaOH and 1.5 M NaCl, washed three times with distilled water and stored at -20°C until use. To ensure that DNA samples were successfully deposited on the membranes, ^33^P-labeled genomic DNA was hybridized to the macroarray before transcriptome analysis.

### Bacterial culture

The *Y. pseudotuberculosis *transcriptome was studied in three independent cultures of strain IP32953 in media aliquoted from a single batch. After storage in Luria-Bertani (LB) broth with 40% glycerol at -80°C, the strain was thawed and then grown on LB agar supplemented with 20 μg ml^-1 ^hemin for 48 h at 28°C. From this culture, 8 × 10^6 ^cells were inoculated into 40 ml of either LB broth or pooled human plasma from healthy donors (heated at 56°C for 30 min to ensure complement inactivation). Media were then incubated at 28°C or 37°C with shaking and *Yersinia *growth was monitored by absorbance at 600 nm.

### RNA and cDNA probe preparation

Cells were harvested from exponential-phase cultures (*A*_600 _of 0.2–0.4 and 0.1–0.2 for LB and human plasma, respectively) by centrifugation at 4°C and the pelleted bacteria were disrupted with RNAwiz reagent (Ambion). After mixing the lysate with chloroform (0.2 v), total RNA was precipitated from the aqueous phase with glycogen (1/50 v) and isopropanol (1 v). The RNA pellet was washed with 70% ethanol and then dissolved in sterile, DNase- and RNase-free water. Contaminating DNA was removed using the DNA-free kit from Ambion. Nucleic acid purity and integrity was checked with a BioAnalyzer 2100 (Agilent) according to the supplier's instructions. After quantification by spectrophotometry at 260 and 280 nm, the RNA solution was stored at -80°C until use. cDNA was further generated from 10 μg of total RNA incubated (in a total volume reaction of 45 μl) for 3 h at 42°C with 50 U AMV reverse transcriptase (Roche), 0.35 pmol. of each amplified CDS-specific 3' oligonucleotide primer, 222 μM dATP, dGTP & dTTP, 2.2 μM dCTP and 50 μCi ^33^P-labelled dCTP (Amersham Biosciences). Labeled cDNA was purified to remove unincorporated nucleotides using DyeEx 2.0 spin column (Qiagen).

### DNA macroarray hybridization

Macroarrays were prewetted in 2 × SSPE (0.18 M NaCl, 10 mM NaH_2_PO_4_, 1 mM EDTA, pH 7.7) and prehybridized for 1 h in 13 ml of hybridization solution (5 × SSPE, 2% SDS, 1× Denhardt's reagent, 0.1 mg of sheared salmon sperm DNA ml^-1^) at 65°C in roller bottles. Hybridization was carried out for 20 h at 65°C with 15 ml of hybridization solution containing the purified cDNA probe. After hybridization, membranes were washed three times at room temperature and three times at 65°C for 20 min in 0.5 × SSPE and 0.2% SDS. Probed macroarrays were exposed to a phosphor screen (Molecular Dynamics) for 24–72 h and imaged using a STORM 860 phosphorimager (Amersham Biosciences). The intensity of all of the pixels associated with each spot was further quantified using ArrayVision software (Imaging Research, Grinnel, IA, USA). The experiment design included three biological replicates for each combination of conditions. Data were analyzed using the SAS software (SAS Institute Inc, Cary, NC, USA). They were first log-transformed and normalized with a median normalization. A linear model was then applied on each gene with the temperature, phase and growth medium as fixed effects. The significance level alpha was set to 0.05.

### Real-Time Quantitative PCR

Messenger RNAs (mRNAs) were reverse transcribed from 1 μg of nucleic acid by using the High-Capacity cDNA Archive Kit (Applied Biosystems, Foster City, CA) according to the manufacturer's instructions. The resulting cDNA was amplified by the SYBR Green Real-Time PCR Kit and detected on a Prism 7000 detection system (Applied Biosystems). The forward and reverse primers used were as follows: 5'CGCCATCAAATGCGCTAAT3' and 5'TGAGCGGGATCGTGTTCAA3' for *yfeA*, 5'TCAAGCAGGGAAACACATTCC3' and 5'GGCTGTTTACCCGCAAAAATC3' for *psaA*, 5'GGTTAGCCGCGAACAGGATA3' and 5'CGCTCGCCAGAACAAGGTT3' for *aceB*, 5'TCGATGCTCGCGCTAAGG3' and 5'GCTGGTTTCGCTGCTTCAG3' for *yadA*, 5'GATCCTGGTTCCATAAAAATTATTCAC3' and 5'ATTGTTCGCCTGGATTACCAA3' for *yopJ*, 5'GAGAATCCCAGTCGGGTGTTAA3' and 5'TCACTGCATCGCGGTAGGT3' for *yopN*, 5'GACACCAGTGGGACGCAACT3' and 5'GGGTTCACAAGAAAGAGTAACAGCTT3' for *sycH*, 5'GGTTACGCGCGGGTATCA3' and 5'CCGCGTCTTTGAGTGTTTTG3' for *tnpR*, 5'TTCTCGTGGGCAACCTATCC3' and 5'TGCGTTCCCAGCATACACAA3' for *nlpD*. On completion of the PCR amplification, a DNA melting curve analysis was performed to confirm the presence of a single amplicon. Relative mRNA levels (2^ΔΔC^) were determined by comparing the PCR cycle thresholds (Ct) for the gene of interest and the constitutively expressed *YPTB0775* gene (spot ID YPO3356) coding for the outer membrane lipoprotein NlpD.

## Abbreviations

qRT-PCR: quantitative Real Time Reverse Transcription PCR.

## Authors' contributions

MLR performed the macroarray hybridizations and participated to the critical proofreading of the manuscript. SC contributed to the experiment set-up and was responsible for bacterial cultures and RNA extractions; she participated in statistical analyses and critical proofreading of the manuscript. RD performed the qPCR experiments. CL, LF, CL, AS, J-YC and CM were involved in the macroarray design and construction. MAD contributed to the experiment design and performed the statistical analyses. JF contributed to the bacterial cultures. EC participated in experimental design and, as the main project coordinator, in critical proofreading of the manuscript. MM contributed to the experimental set-up, performed the spot intensity quantification and the biological interpretation of the results; he wrote this manuscript with assistance of MS, who was also involved in coordination of the project. All the authors have read and approved the content of this article.

## Supplementary Material

Additional file 1 **Validation of macroarray hybridization data.** Transcriptional changes for three chromosomal (*yfeA*, *psaA *and *aceB*) and five plasmid-borne (*yadA*, *yopJ*, *yopN*, *sycH *and *tnpR*) genes (assessed using macroarray hybridization and qRT-PCR assays) are shown. Click here for file
