# Supplementary figures and images for: Growth of Yersinia pseudotuberculosis in human plasma: impacts on virulence and metabolic gene expression
Source: BMC Microbiol. 2008 Dec 3;8:211. doi: 10.1186/1471-2180-8-211 (PMC2631605; doi:10.1186/1471-2180-8-211)

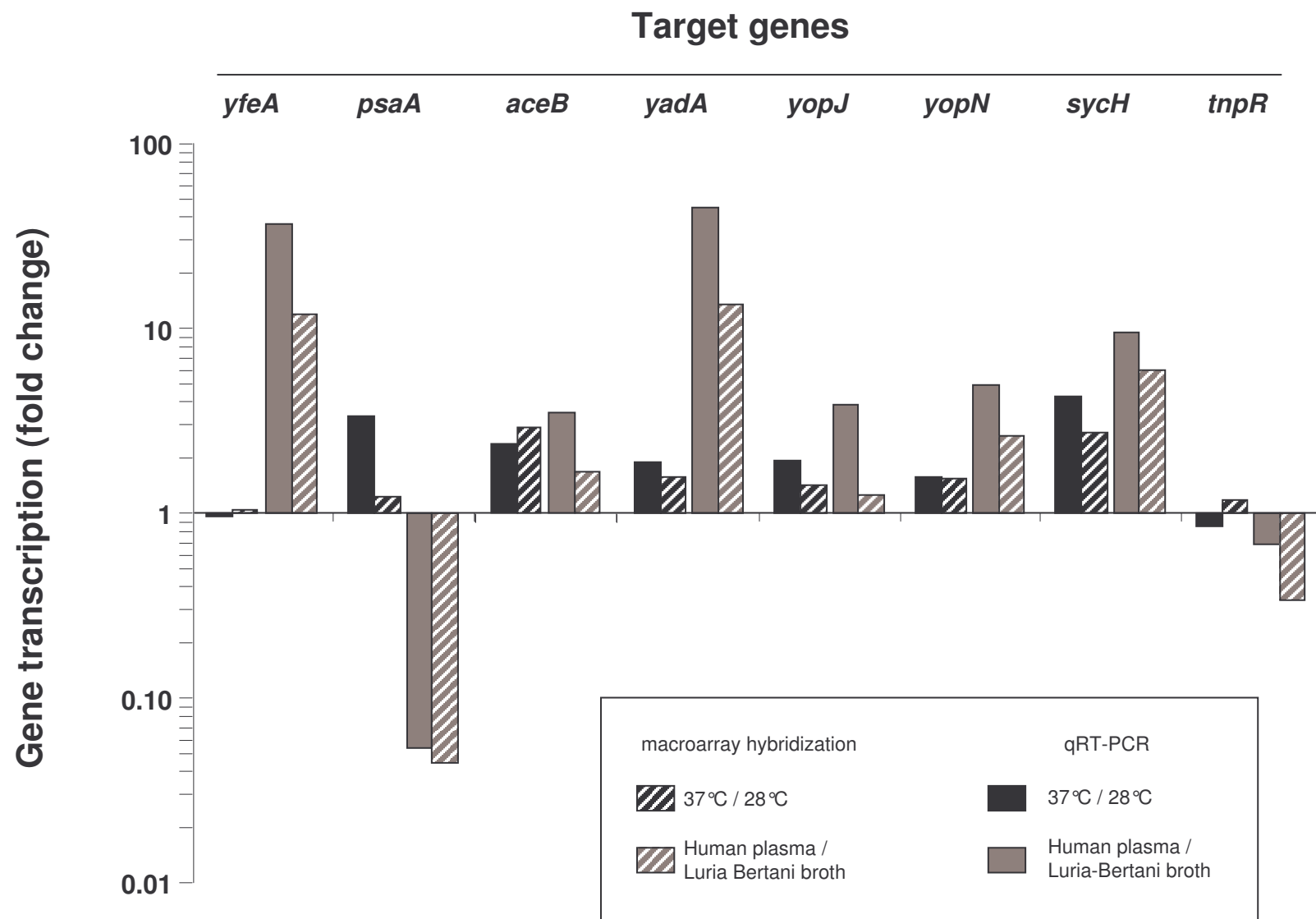

Supplement: Additional file 1 — Validation of macroarray hybridization data. Transcriptional changes for three chromosomal (yfeA, psaA and aceB) and five plasmid-borne (yadA, yopJ, yopN, sycH and tnpR) genes (assessed using macroarray hybridization and qRT-PCR assays) are shown. [file 1471-2180-8-211-S1.pdf]
